# Alchemical Free Energy Estimators and Molecular Dynamics Engines: Accuracy, Precision, and Reproducibility

**DOI:** 10.1021/acs.jctc.2c00114

**Published:** 2022-05-24

**Authors:** Alexander D. Wade, Agastya P. Bhati, Shunzhou Wan, Peter V. Coveney

**Affiliations:** † Centre for Computational Science, Department of Chemistry, University College London, London WC1H 0AJ, UK; ‡ Informatics Institute, University of Amsterdam, Amsterdam 1098XH, The Netherlands; ¶ Advanced Research Computing Centre, University College London, London WC1H 0AJ, UK

## Abstract

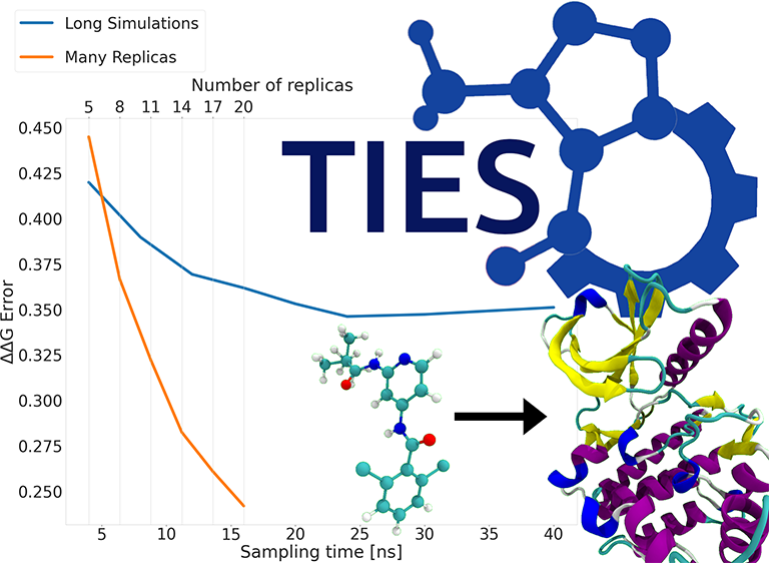

The binding free energy between a ligand and its target protein is an essential quantity to know at all stages of the drug discovery pipeline. Assessing this value computationally can offer insight into where efforts should be focused in the pursuit of effective therapeutics to treat a myriad of diseases. In this work, we examine the computation of alchemical relative binding free energies with an eye for assessing reproducibility across popular molecular dynamics packages and free energy estimators. The focus of this work is on 54 ligand transformations from a diverse set of protein targets: MCL1, PTP1B, TYK2, CDK2, and thrombin. These targets are studied with three popular molecular dynamics packages: OpenMM, NAMD2, and NAMD3 alpha. Trajectories collected with these packages are used to compare relative binding free energies calculated with thermodynamic integration and free energy perturbation methods. The resulting binding free energies show good agreement between molecular dynamics packages with an average mean unsigned error between them of 0.50 kcal/mol. The correlation between packages is very good, with the lowest Spearman’s, Pearson’s and Kendall’s tau correlation coefficients being 0.92, 0.91, and 0.76, respectively. Agreement between thermodynamic integration and free energy perturbation is shown to be very good when using ensemble averaging.

## 1 Introduction

When applied rigorously, computational free energy methods offer the ability to make accurate and precise predictions for protein–ligand binding affinities.^1^ Physics-based free energy methods, while historically being prohibitively expensive, have now become routine, with the development of GPU hardware and GPU-accelerated molecular dynamics (MD) codes.^2−4^ The way in which these calculations are structured provides many opportunities for concurrent execution across high performance computers, allowing predictions for binding affinities including reliable error estimates to be made in the order of hours, a critical time frame in the domains of drug design and personalized medicine.

The accuracy of these calculations has been improved over time as the force fields used to parameterize the system have seen continued development.^5−8^ Ongoing work in the field aims to further these improvements with large collaborative endeavors such as the Open Force Field Initiative^9^ and the development of systematic methods for force field optimization such as Force Balance.^10^ Being able to calculate these binding free energies accurately can be of significant benefit to drug design campaigns, helping reduce the large cost involved with drug development.^11^ Moreover, these calculations can allow much larger areas of chemical space to be explored than would be possible experimentally. Compounds drawn from this chemical space can be selected from numerous sources such as chemical libraries,^12^ repurposing of approved drugs,^13^ generative AI methods,^14−16^ or even other free energy calculations.^17^

Another aspect of MD-based free energy calculations is the extreme sensitivity of such calculations to their initial conditions.^18^ It has been shown that free energies derived from two independent MD simulations, only varying in their starting velocities, can vary by a substantial amount; the exact figure depends on the type of method used and the system studied.^19−26^ MD-based free energy methods require ensemble averaging across the conformations generated. However, the practice has been to perform time averaging over a single trajectory relying on the ergodic theorem, which equates time averaging to ensemble averaging. It is worth mentioning that the ergodic theorem holds true only in the limit of infinite time, which is far from the typical length of simulations performed. This explains the observed differences in free energies between repeat simulations. Indeed, a recent study showed that ensembles are required to handle both aleatoric and parametric uncertainty in MD simulation.^27^ It has also been shown that running an ensemble of independent simulations varying only in their starting conditions or, in other words, an ensemble simulation yields precise and reproducible results.^21,25^ In particular, methods such as ESMACS^21,22^ and TIES^25,26^ have been developed based on such ensemble simulations so as to ensure reproducibility and hence reliability of the predicted free energies. Recently, we have also shown computationally and experimentally that the distributions of free energies obtained from such ensemble simulations are in general not Gaussian as is the common assumption; rather, they exhibit non-normality, which has interesting and important consequences.^28−31^

Alchemical binding free energy calculations are a class of free energy methods that involve the transformation of one or more chemical moieties in the system to another.^32^ Alchemical protein–ligand binding calculations can be performed in an absolute or relative fashion.^33,34^ In an absolute calculation, the binding free energy of a ligand is calculated by completely removing the ligand from the protein–ligand complex. Alternatively, one can perform relative calculations that compare the binding free energy between two ligands. During a relative calculation, one ligand is transformed, via unphysical “alchemical” intermediate states, into another. The two ligands studied generally have a highly conserved chemical structure; this is both a strength and a weakness of the method since the practitioner is restricted to studying cogeneric ligands but benefits from potential gains in accuracy and precision resulting from studying smaller changes when compared to absolute methods. Cogeneric ligands also come with some other tangential benefits, such as avoiding complicating factors involving standard state corrections.^35,36^ In this study, we have employed the ensemble simulation-based TIES^25^ to correctly handle the uncertainties associated with such calculations and extended this to apply to free-energy perturbation methods.

In this work, we consider only relative binding free energy (RBFE) calculations. Several existing software applications can facilitate these calculations such as PMX based on GROMACS,^37^ FEP+ proprietary software produced by Schrödinger,^38^ or FESetup.^39^ Our group has recently publicly released the comprehensive TIES toolkit^40^ to automatically setup, execute, and analyze such calculations; this software was used to prepare and perform all calculations for this study. The specifics of RBFE methodologies vary between implementations, being based on user choices about how to carry out calculations. Some key areas where this variation could significantly influence the results include the topology of the transformed moieties, the thermodynamic path followed between end states, and how much sampling is performed in each state. These factors introduce some uncertainty in the results, but this is generally controlled by probing them on a case by case basis.

For the application of RBFE calculations to the protein-ligand binding problem, one aspect of uncertainty quantification which has received less attention is the variation in results across MD packages. In previous work by Rizzi *et al*., a wide array of alchemical methods were compared, including the potential of mean force and weighted ensemble methods.^41^ This study reported that the variability in the absolute binding free energy across the methods tested is in the range of 0.3 to 1.0 kcal/mol. However, due to differences between the methods tested, comparisons are difficult to draw across alchemical methods or estimators. Moreover, unlike our current study, that study does not directly compare the performance of different MD packages using the same alchemical methods, which adds too many variables for systematic and direct comparisons to be made. The input systems used by Rizzi *et al*. were closely matched but with some differences arising from factors such as different Coulomb constants used by AMBER and CHARMM, differing implementations of particle–mesh Ewald methods or Lennard–Jones (LJ) cutoff schemes. Technical differences between MD codes are a recurring issue, which complicates the comparison of calculations and plays an important role in the present study.

Another study that proposes a comparison between estimators using some simple benchmark systems has been carried out previously by Paliwal *et al*.,^42^ who studied in detail the properties of numerous perturbative estimators as well as thermodynamic integration. All estimators are examined using GROMACS, allowing meaningful comparisons to be made. However, the systems used by Paliwal *et al*. are small toy models and, hence, are not relevant for larger protein–ligand systems as used in this work. One of the ways in which uncertainty is quantified in their work was to run an ensemble of 100 simulations and calculate the mean and standard deviation of the binding free energies from each simulation. Using large ensembles allowed Paliwal *et al*. to quantify the type of distribution for calculated hydration free energies. From the calculated binding free energy distributions, it is concluded that the assumptions of Gaussian distributed errors in free energies are usually valid for most methods studied. This is contrary to the observations made in our work where, when using the same free energy estimators for an investigation of more complex protein-ligand systems, it is found that Gaussian distributions cannot be assumed as also reported in some of our previous studies.^28,29^

In the present paper, we investigate the reproducibility of relative binding free energy calculations using three MD packages and two free energy estimators. The use of ensemble-based simulations will be made to control uncertainties as is essential for any calculation reliant on chaotic MD trajectories. Using ensembles to provide robust error control, we aim to identify statistically significant differences in the results from the different MD packages and estimators.

## 2 Theory

In this section, we outline the essential theory underpinning the alchemical methods we study.

### 2.1 Alchemical Methods

Applied to protein–ligand binding problems, alchemical methods involve changing chemical moieties in the studied system and calculating the free energy differences associated with these changes. Since in atomistic simulations, systems are parameterized by force fields, the transformation of chemical moieties can be achieved by modifying the atomistic parameters of the system. The variable λ is designated to control the modified parameters of the system, turning on and off relevant inter- and intramolecular potentials. The reduced potential *u*(***x***, λ) of such a system can therefore be written as a function of the controlling parameter
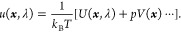
1

Here, ***x*** is the configuration of the system, *U* is the potential energy, *p* is the pressure, and *V* is the volume, plus any other terms relevant to the specific ensemble in which the simulation is performed e.g., NPT, grand, etc. As an example, consider the transformation of some ligand A to some ligand B. The value of λ ranges between 0 and 1; with a λ of 0, the system is in a state describing ligand A, and with a λ of 1, the system is in a state describing ligand B. Typically, λ will take multiple intermediate values between the end states 0 and 1; this range of λ values λ_0_, λ_0.1_, λ_0.2_...λ_1_ defines a set of alchemical states, and simulations are performed in all these states. The choice of these states is not arbitrary and can affect both the accuracy and precision of the results.

### 2.2 Thermodynamic Integration

To calculate free energy differences with alchemical methods one of many available estimators can be used. In this work, an application of a thermodynamic integration (TI) estimator is made with enhanced sampling (TIES^25^); this methodology has been used in numerous studies to calculate accurate and precise RBFEs.^25,26,29^ Centrally, TIES is based on the formally exact TI equation
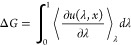
2

Here, *G* is the Gibbs free energy and Δ*G* is the change in Gibbs free energy between two states A and B. Δ*G* is calculated by integrating over the range of λ, and this integration is performed numerically. It is worth highlighting that eq 2 is only strictly valid in the thermodynamic limit when both left-hand-side and right-hand-side terms are unique numbers with no fluctuations. However, practically speaking, we work with finite systems and sample only a fraction of the full conformational space, which makes these quantities stochastic variables.^18,28^ This implies that both the free energy as well as its derivative will have a distribution of values. Therefore, it is necessary to get the expectation value of these quantities using ensemble methods. The brackets ⟨. ⟩_λ_ denote an ensemble average in a thermodynamic state defined by the value of λ. To compute this ensemble average, the configurations of particles can be sampled using Monte Carlo or MD methods, and from these sampled configurations, values of the potential are calculated and averaged. The traditional approach has been to perform a single “long” MD simulation to proxy ensemble averaging with time averaging. However, as discussed already, this is not reliable due to the extreme sensitivity of the results obtained, arising from the initial conditions that are controlled by the random seeds used to initiate simulations. Thus, ensemble simulations are required to generate the ensemble of conformations in order to estimate an average and distribution of the calculated Δ*G*. In this work, the same idea of performing ensemble simulation to get the expectation value of the distribution of Δ*G* by performing stochastic integration of the distributions of  is applied using the TIES methodology.

### 2.3 Free Energy Perturbation

Parallel to the set based on TI are perturbative methods such as free energy perturbation (FEP) methods. The simplest estimators belonging to this class of perturbative methods are those based on the Zwanzig relation. However, it is known in FEP calculations that free energy estimates from the Zwanzig relation can be prone to bias stemming from the dominant contribution of rare samples when using finite sampling.^43^ As such, there exist several methods that aim to improve the exponential averaging estimator; these are the Bennet acceptance ratio (BAR) and the multistate Bennet acceptance ratio (MBAR). In this work, the MBAR estimator is used, the derivation of this method is given in detail in the work of Shirts and Chodera.^44^ Here, we present the relevant equation from this previous work for computing dimensionless free energies in a system with *K* total λ states,
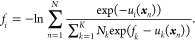
3

In this equation, *f*_*i*/*k*_ is the dimensionless free energy for the state with λ = λ_*i*/*k*_, *N* is the total number of samples indexed by *n*, *N_k_* is the number of samples collected in state λ = λ_*k*_, and *u*_*i*/*k*_(***x***_*n*_) is then the reduced potential energy evaluated in state λ = λ_*i*/*k*_ calculated using the configuration sampled in iteration *n*. Note that the summations run over all alchemical windows, and thus information from all windows is combined to produce a free energy estimate; if only two windows are considered, MBAR reduces to BAR.^44^ This equation can be solved self-consistently with many solvers, and these methods are implemented in the pymbar package,^44^ which was used in this work to compute results with MBAR. The dimensionless free energies in eq 3 are combined into free energy differences and converted to the Gibbs free energies as follows
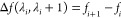
4
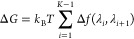
5

If the overlap in phase space between adjacent alchemical states is low, it can be difficult to sample sufficiently to calculate trustworthy free energy differences with FEP methods.^45^ No rigorous criteria exists that relates the expected variance in the calculated free energy to the amount of sampling or overlap between states for a given system. As a result, there are numerous other ways in which the reliability of FEP calculations are tested,^43,45^ such as calculating the convergence of results with the amount of sampling/number of alchemical windows or computing overlap distributions and overlap matrices.^45^ The main way in which the variance in FEP calculation will be addressed in this work is through the use of ensembles of simulations. As described above for the TI estimator, the concept of ensemble simulations to obtain the expectation value of Δ*G* along with associated uncertainty will also be applied to FEP.

### 2.4 Ligand Protein Binding Free Energy

Δ*G* can be calculated with many different estimators. In order to calculate the binding free energy difference of ligand to protein, ΔΔ*G*, calculated values for Δ*G* are combined through a thermodynamic cycle.^33^ In the case of RBFE for protein ligand binding the following thermodynamic cycle is routinely used
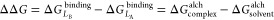
6here Δ*G*_solvent/complex_^alch^ are the Δ*G*s calculated in eqs 2 and 4 in the solvent/complex simulations (transforming *L*_A_ into *L*_B_). Where the solvent simulation is the ligand in solvent and the complex simulation is the ligand in complex with the solvated protein. Δ*G*_*L*_A_/*L*_B__^binding^ is the binding free energy of ligand A/B to the protein. The difference of these alchemical free energies is equal to the difference of binding free energy of the ligands A and B, which allows the final ΔΔ*G* to be calculated.

## 3 Methods

The RBFE calculations performed in this work are calculated using three molecular dynamics packages; these are OpenMM, NAMD 2, and NAMD 3 alpha. OpenMM can perform MD calculations on multiple platforms (CPU, CUDA, and OpenCL); in this work, all OpenMM calculations are performed using the CUDA platform with OpenMM 7.4.2. Likewise, NAMD3 alpha calculations are run on CUDA GPUs. The NAMD2 calculations are performed on CPUs.

To automate the setup and running of these simulations, we have developed and released an open-source Python package called TIES MD, which is available online^1^. This study uses the existing input files from previous research that works with TIES MD; novel input ligand transformations can be generated using an online service or open-source installable package TIES 20^2^. The combination of TIES MD and TIES 20 allows anyone to freely and easily use the TIES protocol to calculate binding free energies.

### 3.1 Input Systems

All the methods in this work use the same dual topology input systems. These systems model 5 proteins and 54 ligand transformations. The models are taken from the previous work of Bhati *et al*.,^25^ and details of their preparation are provided in that paper. In the SI of this paper, we provide all these parametrized systems, and note here that the AMBER ff99SB-ILDN^46^ force field was used for protein parameters and the ligand parameters were produced using the general AMBER force field (GAFF).^47^

### 3.2 Simulation Protocol

The number of input parameters to MD engines is large (175 in the case of NAMD2); matching these between engines is challenging and conceivably an obstacle to the reproducibility of results. Recent work by Vassaux *et al*. examining the parametric uncertainty of NAMD2^27^ has shown that only six input parameters dominate the error in free energy calculations. Moreover, it is clear from the work of Vassaux *et al*. that the parametric uncertainty is damped in the output of free energy calculations. Combined with the corpus of literature that shows that aleatoric error is dominating in MD simulations,^27,30^ the parametric differences are of less concern and should not impede reproducibility.

Our general alchemical protocol involves collecting samples from 13 intermediate alchemical states. This entails running an energy minimization followed by 2 ns of equilibration. After running pre-production on each state, 4 ns of NPT production sampling is performed. In each state, an ensemble of five simulations is performed for each simulation leg to calculate one Δ*G* value. From the production sampling the potential and gradient, , are calculated every 4 ps.

### 3.3 OpenMM Alchemical Protocol

The molecular dynamics sampling in OpenMM was performed using NVT and NPT ensembles. In the NVT ensemble, Langevin dynamics was used with a friction coefficient of 300 fs, a target temperature of 300 K, and an integration time step of 2 fs. In the NPT ensemble, a Monte Carlo barostat was added with pressure changing moves attempted every 25 steps and a target pressure of 1 atm. A nonbonded cutoff of 1.2 nm was used with a switching distance of 1.0 nm. Any long-range dispersion corrections are turned off for parity with NAMD calculations. The particle mesh Ewald (PME) algorithm was used to calculate the electrostatic contribution to the potential; this was performed with an error tolerance of 0.00001. OpenMM computed the number of nodes in the PME mesh dependent on the nonbonded cutoff, error tolerance, and size of the simulation cell.^3^ Preproduction of the OpenMM calculation involved a constrained minimization using OpenMM’s implementation of the limited memory Broyden–Fletcher–Goldfarb–Shanno algorithm. This was followed with 20 ps of NVT equilibration and then 2 ns of NPT equilibration. After this, 4 ns of NPT production was performed with samples of the potential and gradient, , collected every 4 ps.

OpenMM does not offer any inbuilt alchemical methods, and as such, there exist a number of programs that extend OpenMM, allowing systems to be manipulated alchemically and perform alchemical calculations. One such program used in this work, is OpenMMTools0.19.0.^48^ OpenMMTools can take as input a standard OpenMM system, defined with some potentials, and transform this system into an alchemical one, where the potentials are controlled by the λ parameter. The scaling of (LJ) interactions was performed with a soft-core potential using the functional form of eq 13 presented in the work of Pham *et al*.^49^ with the following parameters: α = 0.5, *a* = 1, *b* = 1 and *c* = 6, the default parameters used by OpenMMTools. Electrostatic interactions are scaled linearly without a soft-core potential. The λ schedule used in the OpenMM calculations was a two-step procedure, which completely annihilated all electrostatic interactions of outgoing alchemical moieties before scaling down the LJ interaction and completely created all LJ interactions of incoming moieties before turning on any electrostatic interactions. Annihilation was used in the OpenMM method as this is the methodology supported by OpenMMTools when calculating the electrostatics with the PME method, which was used for all simulations in this work. In this context, annihilation means that when a chemical moiety is “turned off,” both inter- and intramolecular interactions are extinguished.

### 3.4 NAMD2 Alchemical Protocol

The molecular dynamics sampling in NAMD was performed using NVT and NPT ensembles. For NAMD NVT, sampling is collected using Langevin dynamics with a friction coefficient of 500 fs, a target temperature of 300 K, and an integration time step of 2 fs. In NAMD2 calculations, a Berendsen barostat was used with a compressibility of 4.57 × 10^–5^bar^–1^, relaxation time of 100 fs, and target pressure of 1 atm. A nonbonded cutoff of 1.2 nm was used with a switching distance of 1.0 nm. A pair list distance of 1.35 nm is used with an update frequency of 20 steps. No long-range dispersion corrections are applied. The PME algorithm was used to calculate the electrostatic contribution to the potential; this was performed with an error tolerance of 0.000001 and a PME grid spacing of 0.1 nm. Preproduction of the NAMD calculation involved a constrained minimization using NAMD’s implementation of the conjugate gradient method. This was followed with 20 ps of NVT equilibration and then 2 ns of NPT equilibration. After this, 4 ns of NPT production was performed with samples of the potential and gradient, , collected every 4 ps.

The NAMD method uses a soft-core potential to decouple the LJ interactions. This soft-core potential can be expressed in the same form as the OpenMM soft-core using parameters α = 0.5, *a* = 1, *b* = 1 and *c* = 2, the default parameters used by NAMD. Electrostatic interactions are decoupled linearly without a soft-core potential. The λ schedule used in the NAMD calculations was a one-step procedure where LJ and electrostatic potentials are scaled simultaneously but at a different pace. Decoupling was used in the NAMD method as this is the method invoked in our original NAMD2-based study of these systems.^25^ In this context, decoupling means that when a chemical moiety is “turned off,” only the intermolecular interactions are removed.

While this procedure describes the simulation protocol accurately, one caveat must be added in the case of the NAMD2 results. The results presented here for NAMD2 are from the work of Bhati *et al*.^25^ In this previous work, the gradients used in eq 2 are collected at intervals of 4 ps, but the trajectories are saved at intervals of 10 ps. Therefore, the post-processing of FEP results can only be calculated at intervals of 10 ps. This affects the comparison of TI and FEP results, and we address the matter as it arises in the analysis of the results.

### 3.5 NAMD3 Alchemical Protocol

At the time of writing, there was not perfect feature parity between NAMD2 and NAMD3 alpha; thus, NAMD3 used a different barostat for NPT simulations. In NAMD3, a Langevin piston was used with a piston period of 200 fs and a piston decay of 100 fs. With the exception of the barostat, all settings are the same between NAMD2 and NAMD3.

### 3.6 Uncertainty Quantification

For the FEP estimator-based results presented in this work, each one of the replicas in the ensemble of five simulated allowed for the calculation of one Δ*G* by applying eq 3 to the potentials sampled from the simulation. The five resulting values of Δ*G* are then bootstrapped to calculate a mean and standard error of the mean (SEM). For the TI results, we apply the TIES protocol as it has been used in previous work.^25^ The defining characteristic of TIES is the use of an ensemble of simulations in each alchemical state to control the aleatoric errors inherent to MD simulations. In every one of the total 13 alchemical states, an ensemble of five simulations is performed, each of which yields a time series of , which can be averaged to give . An ensemble of five such values is then bootstrapped to calculate the mean, which is used as the final value in eq 2. Each bootstrapping provides an estimate in the uncertainty as a SEM of the gradient in each alchemical window, σ^2^(λ), which is propagated as follows to give a total estimate of the uncertainty in each Δ*G* calculation
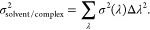
7

Here, σ_solvent/complex_^2^ is the variance in one thermodynamic leg of the simulation and Δλ is the difference between the value of λ between adjacent windows. Errors from complex and solvent legs are combined in quadrature for both TIES and FEP methods to calculate the final uncertainty on the binding free energy.

### 3.7 Performance

Our simulations were run across several high performance computers including Summit at the Oak Ridge National Laboratory, ThetaGPU at the Argonne Leadership Computing Facility, SuperMUC-NG at the Leibniz Supercomputing Centre, and ARCHER2, the UK’s national high-performance computer service. The performance of OpenMM is calculated while running on one Nvidia V100 GPU with a ligand–protein complex of 35k atoms, which achieves simulation speeds of 115 ns/day. The performance of NAMD3 is calculated while running on one Nvidia A100 GPU with a ligand–protein complex of 35k atoms, which achieves simulation speeds of 145 ns/day. Therefore, a TIES calculation on GPU using 13 alchemical windows and 5 replica simulations per window takes around 60 min of wall time using 65 V100/A100s. NAMD2 is performed on a CPU platform and using 96 Xenon Skylake cores; the simulation speed is 26 ns/day. Thus, using NAMD2 and 6240 cores for one TIES calculation, again with 13 windows and 5 replicas, takes around 5–6 h of wall time on the CPU. In OpenMM, the calculation of potentials and gradients required for FEP and TI analysis can be performed concurrently with the simulation; this creates an overhead of around 10% in TIES MD. The speed of OpenMM without this overhead is therefore 127 ns/day. For our NAMD calculations, either the potential or gradient can be saved with the simulation but not both. Therefore, the TI and FEP results cannot be collected concurrently, and a post-processing step is needed to extract the FEP result from the NAMD trajectories. This post-processing generally takes 10–20 min for one TIES calculation. More detail of the performance of NAMD and OpenMM codes is provided in the Supplementary Information.

## 4 Results

In this section, we present the wide range of results obtained in this study, covering comparisons between MD packages and free energy protocols, ensembles versus one-off simulations, and the free energy distributions found.

### 4.1 Comparing Molecular Dynamics Engines

In the present work, we study 54 ligand transformations in the protein targets MCL1, PTP1B, TYK2, CDK2, and thrombin. Here, we present the results of these calculations, comparing accuracy and precision across the MD packages and free energy estimators. Tables 1 and 2 present a comparison of the accuracy and precision of all methods compared to those of the experiment.

**Table 1:** Statistical Properties Calculated for all Protein Targets and MD Engines Using TI^a^

protein	property	NAMD3 TI	NAMD2 TI	OpenMM TI
PTP1B	MUE	0.63[0.36, 1.09]	0.48[0.26, 0.70]	0.61[0.41, 0.79]
	MSE	0.71[0.15, 1.66]	0.35[0.16, 0.60]	0.46[0.26, 0.74]
	RMSD	0.85[0.40, 1.31]	0.59[0.40, 0.78]	0.68[0.51, 0.86]
	Person’s	0.44[−0.17, 0.88]	0.68[−0.59, 0.87]	0.36[−0.45, 0.73]
	slope	0.37[−0.01, 1.03]	0.65[0.17, 1.30]	0.70[−1.15, 2.15]
	intercept	0.31[−0.32, 0.84]	0.13[−0.59, 0.85]	0.31[−0.89, 0.83]
CDK2	MUE	0.94[0.55, 1.45]	0.76[0.38, 1.07]	0.98[0.62, 1.66]
	MSE	1.23[0.54, 2.73]	0.78[0.34, 1.27]	1.41[0.52, 3.64]
	RMSD	1.11[0.73, 1.67]	0.89[0.56, 1.13]	1.19[0.73, 1.92]
	Person’s	0.87[0.53, 0.97]	0.83[−0.07, 0.94]	0.89[0.71, 0.96]
	slope	0.48[0.26, 0.79]	0.57[0.31, 1.14]	0.46[0.26, 0.72]
	intercept	0.09[−0.42, 0.53]	0.01[−0.75, 0.46]	0.18[−0.24, 0.56]
MCL1	MUE	1.36[1.03, 1.73]	1.17[0.86, 1.51]	0.98[0.63, 1.66]
	MSE	2.36[1.48, 3.66]	1.82[1.07, 2.95]	1.82[0.70, 4.96]
	RMSD	1.54[1.21, 1.92]	1.35[1.03, 1.70]	1.35[0.85, 2.22]
	Person’s	0.80[0.54, 0.93]	0.81[0.59, 0.92]	0.74[0.31, 0.92]
	slope	0.52[0.37, 0.65]	0.56[0.38, 0.74]	0.70[0.31, 0.99]
	intercept	–0.09[−0.56, 0.40]	–0.05[−0.51, 0.41]	–0.42[−0.82, 0.09]
TYK2	MUE	0.68[0.48, 0.88]	0.42[0.22, 0.66]	0.62[0.42, 0.94]
	MSE	0.58[0.34, 0.92]	0.31[0.12, 0.62]	0.55[0.27, 1.26]
	RMSD	0.76[0.59, 0.96]	0.56[0.34, 0.79]	0.74[0.53, 1.12]
	Person’s	0.89[0.72, 0.96]	0.94[0.83, 0.99]	0.89[0.74, 0.95]
	slope	0.93[0.65, 1.29]	1.12[0.84, 1.36]	1.05[0.71, 1.56]
	intercept	0.22[−0.39, 0.82]	0.15[−0.30, 0.59]	0.11[−0.47, 0.73]
thrombin	MUE	0.98[0.69, 1.31]	0.63[0.43, 0.79]	0.85[0.66, 1.12]
	MSE	1.25[0.74, 2.30]	0.49[0.29, 0.72]	0.87[0.51, 1.46]
	RMSD	1.12[0.87, 1.50]	0.70[0.55, 0.85]	0.93[0.72, 1.22]
	Person’s	0.87[0.65, 0.96]	0.92[0.81, 0.98]	0.89[0.62, 0.96]
	slope	0.47[0.36, 0.65]	0.59[0.49, 0.77]	0.49[0.38, 0.61]
	intercept	–0.10[−0.50, 0.24]	–0.02[−0.34, 0.21]	0.14[−0.15, 0.42]

a Properties are calculated with comparison to experimental data. Properties and 95% confidence intervals, provided in square brackets, are calculated with bootstrapping. All energies are in kcal/mol.

**Table 2:** Statistical Properties Calculated for all Protein Targets and MD Engines Using FEP^a^

protein	property	NAMD3 FEP	NAMD2 FEP	OpenMM FEP
PTP1B	MUE	0.59[0.27, 1.11]	0.36[0.21, 0.50]	0.60[0.43, 0.74]
	MSE	0.73[0.09, 1.79]	0.18[0.08, 0.28]	0.42[0.25, 0.60]
	RMSD	0.85[0.29, 1.33]	0.43[0.29, 0.54]	0.65[0.50, 0.78]
	Person’s	0.44[−0.13, 0.94]	0.83[0.42, 0.95]	0.48[−0.22, 0.82]
	slope	0.38[−0.07, 1.10]	0.80[0.32, 1.21]	0.96[−0.48, 2.50]
	intercept	0.27[−0.22, 0.93]	0.02[−0.39, 0.63]	0.23[−0.88, 0.80]
CDK2	MUE	0.94[0.52, 1.39]	0.76[0.38, 1.07]	0.98[0.56, 1.70]
	MSE	1.23[0.59, 2.67]	0.78[0.34, 1.27]	1.48[0.52, 3.96]
	RMSD	1.11[0.76, 1.67]	0.89[0.56, 1.13]	1.22[0.72, 1.99]
	Person’s	0.87[0.55, 0.97]	0.83[−0.07, 0.94]	0.88[0.68, 0.95]
	slope	0.48[0.27, 0.79]	0.57[0.31, 1.14]	0.45[0.25, 0.75]
	intercept	0.08[−0.47, 0.51]	0.01[−0.75, 0.46]	0.17[−0.29, 0.58]
MCL1	MUE	1.29[0.98, 1.66]	1.22[0.86, 1.69]	0.97[0.64, 1.64]
	MSE	2.15[1.30, 3.45]	2.23[1.21, 4.21]	1.80[0.68, 5.05]
	RMSD	1.47[1.14, 1.88]	1.49[1.09, 2.07]	1.34[0.82, 2.29]
	Person’s	0.82[0.57, 0.93]	0.81[0.55, 0.92]	0.72[0.23, 0.91]
	slope	0.54[0.38, 0.67]	0.53[0.36, 0.72]	0.68[0.27, 1.03]
	intercept	–0.11[−0.59, 0.35]	–0.12[−0.58, 0.32]	–0.34[−0.75, 0.19]
TYK2	MUE	0.66[0.47, 0.85]	0.38[0.20, 0.63]	0.63[0.45, 0.91]
	MSE	0.54[0.32, 0.90]	0.27[0.11, 0.54]	0.53[0.29, 1.19]
	RMSD	0.74[0.57, 0.94]	0.52[0.33, 0.73]	0.73[0.53, 1.09]
	Person’s	0.90[0.75, 0.97]	0.95[0.85, 0.99]	0.89[0.71, 0.95]
	slope	0.95[0.65, 1.29]	1.11[0.84, 1.33]	1.03[0.70, 1.54]
	intercept	0.22[−0.37, 0.79]	0.11[−0.33, 0.51]	0.12[−0.44, 0.73]
thrombin	MUE	0.87[0.53, 1.24]	0.68[0.44, 0.88]	0.82[0.63, 1.07]
	MSE	1.12[0.56, 1.93]	0.60[0.34, 0.90]	0.81[0.47, 1.29]
	RMSD	1.06[0.76, 1.41]	0.78[0.59, 0.96]	0.90[0.69, 1.13]
	Person’s	0.91[0.71, 0.96]	0.92[0.74, 0.97]	0.89[0.58, 0.96]
	slope	0.48[0.38, 0.61]	0.55[0.45, 0.68]	0.50[0.40, 0.62]
	intercept	–0.07[−0.36, 0.20]	0.04[−0.24, 0.27]	0.10[−0.18, 0.39]

a Properties are calculated with comparison to experimental data. Properties and 95% confidence intervals, provided in square brackets, are calculated with bootstrapping. All energies are in kcal/mol.

**Figure 1:**
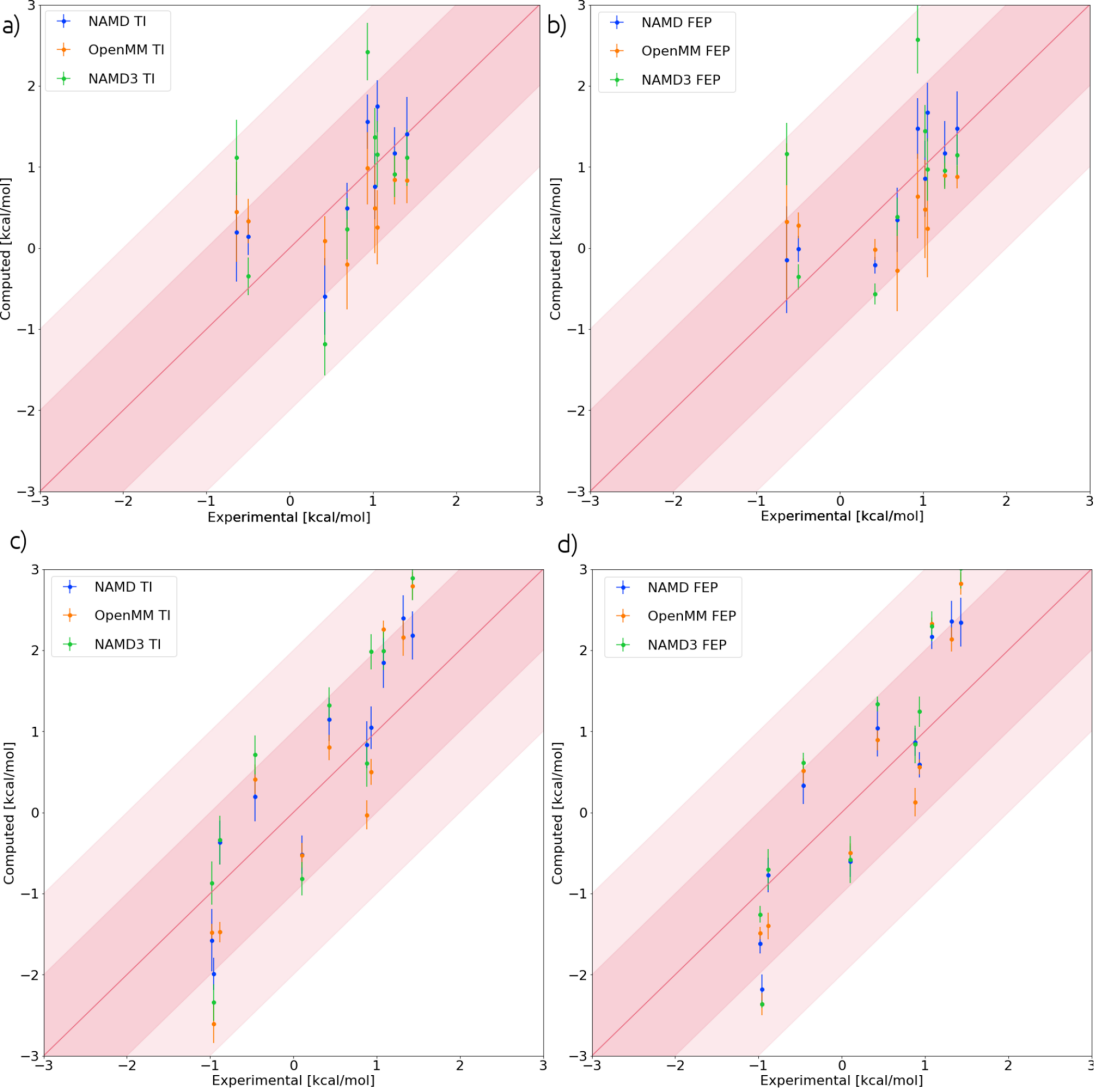
Computed vs experimental ΔΔ*G* values. (a) and (b) show the PTP1B results, and (c) and (d) show the thrombin results. (a) and (c) use TI panels; (b) and (d) use FEP. Computed ΔΔ*G* and errors are SEM from an ensemble of replicas. The dark shaded region spans ±1 kcal/mol; the lighter region spans ±2 kcal/mol.

In Tables 1 and 2, it can be seen that the results across all MD packages and estimators agree well with one another. With 95% confidence intervals, the only cases for which a statistically significant difference can be observed are for the PTP1B and thrombin target. For PTP1B, the methods NAMD2 FEP and OpenMM FEP have MUE, MSE, and RMSD that differ by 0.24(0.23), 0.23(0.20), and 0.22(0.19) kcal/mol, respectively. For thrombin, the MUE, MSE, and RMSD of NAMD2 TI and NAMD3 TI differ by 0.36(0.34), 0.76(0.56), and 0.42(0.30) kcal/mol, respectively. To investigate the PTP1B and thrombin cases, further plots are presented in Figure 1 for the PTP1B and thrombin results compared to the experiment.

From Figure 1, it can be seen that when comparing individual ΔΔ*G*s and using the SEM error, there are some statistically significant differences between methods. Note that in Figure 1, there are no error bars on the *x* axis; this is because no errors are reported with the experimental results.^25^ The limited number of differences should not detract from the overall excellent agreement between all other cases and methods; in fact, some difference in the results from different MD packages should be expected due to the unavoidable differences in implementation detailed in the Methods section and the reasonable probability that some values disagree within 1 standard deviation of error. The difference in individual ΔΔ*G* calculated with different MD packages and free energy estimators is shown in Table 3, where the averaged MUE between methods is 0.50 kcal/mol. Due to the number of differences between methods highlighted in previous sections, it is not possible to comment on what precisely causes any particular difference here. Despite some differences for individual ΔΔ*G* calculations in MD packages, overall, the results are well reproduced. This can also be seen from the properties calculated in Table 3, where the rank order coefficients indicate a strong correlation between all methods with the lowest Spearman’s, Pearson’s, and Kendall’s correlation coefficients between two packages being 0.92[0.89, 1.00] , 0.91 [0.87, 0.95], and 0.76 [0.69, 0.84], respectively.

**Table 3:** Statistical Properties Measuring the Agreement between ΔΔ*G*s Calculated from Different MD Packages in the TI and FEP Cases^a^

estimator	property	OpenMM/NAMD2	OpenMM/NAMD3	NAMD2/NAMD3
TI	MUE	0.51 [0.38, 0.64]	0.58 [0.44, 0.71]	0.49 [0.38, 0.59]
	MSE	0.49 [0.26, 0.69]	0.61 [0.30, 0.87]	0.38 [0.21, 0.53]
	RMSD	0.70 [0.55, 0.86]	0.78 [0.61, 0.97]	0.62 [0.49, 0.75]
	Spearman’s	0.92 [0.89, 1.00]	0.92 [0.89, 0.99]	0.96 [0.93, 1.01]
	Pearson’s	0.91 [0.87, 0.95]	0.92 [0.88, 0.97]	0.95 [0.93, 0.98]
	Kendall’s	0.77 [0.69, 0.85]	0.76 [0.69, 0.84]	0.84 [0.78, 0.91]
	slope	0.86 [0.72, 0.98]	1.10 [0.96, 1.23]	1.08 [0.96, 1.19]
	intercept	0.04 [−0.16, 0.24]	0.04 [−0.16, 0.26]	0.04 [−0.13, 0.20]
FEP	MUE	0.49 [0.38, 0.60]	0.51 [0.37, 0.64]	0.42 [0.33, 0.50]
	MSE	0.41 [0.21, 0.58]	0.53 [0.23, 0.77]	0.29 [0.17, 0.38]
	RMSD	0.64 [0.51, 0.78]	0.73 [0.55, 0.92]	0.54 [0.44, 0.64]
	Spearman’s	0.95 [0.93, 1.00]	0.94 [0.92, 0.99]	0.96 [0.94, 1.00]
	Pearson’s	0.93 [0.90, 0.95]	0.93 [0.9, 0.96]	0.96 [0.94, 0.99]
	Kendall’s	0.79 [0.73, 0.86]	0.79 [0.73, 0.86]	0.84 [0.78, 0.91]
	slope	0.86 [0.72, 0.97]	1.11 [0.97, 1.23]	1.07 [0.98, 1.16]
	intercept	0.00 [−0.18, 0.19]	0.06 [−0.12, 0.27]	0.02 [−0.12, 0.15]

a Properties and 95% confidence intervals, provided in square brackets, are calculated with bootstrapping. All energies are in kcal/mol.

### 4.2 Comparing Free Energy Estimators

**Figure 2:**
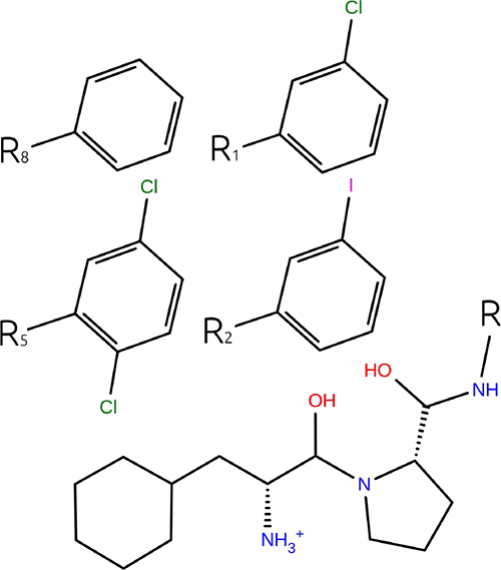
Labeled ligand transformations for which TI and FEP yield a different result in NAMD2 TI and FEP methods. Moieties labeled *R_x_* are substituted onto the common substructure at the position denoted by *R*; e.g., swapping *R*_1_ and *R*_8_ is the ligand transformation l1-l8.

A key result from Tables 1 and 2 is that there is no statistically significant difference between the calculated properties for TI and FEP results in all cases. In order to make the comparison between FEP and TI more rigorous, the calculated ΔΔ*G*s for each ligand transformations are compared individually by taking the difference of the TI and FEP result and calculating the error on this difference by adding the TI and FEP SEM in quadrature. From this comparison, six transformations are identified as having significantly different TI and FEP results. All differences are found for the thrombin and MCL1 targets when using the NAMD methods. The OpenMM implementation had no significant differences for any protein targets. In the NAMD2 case, the significantly different transformations are for thrombin l1-l8 and l2-l5 and for NAMD3 thrombin l2-l5, l1-l4, l4-l11, and MCL1 l3-l16. These transformations are named in the work of Bhati et al.,^25^ and Figure 2 shows the selected NAMD2 ligand transformations explicitly.

#### 4.2.1 Soft-Core Potentials in TI Calculations

All the transformations in Figure 2 feature the transformation of one phenyl group containing a halogen atom. From this similarity, it might be concluded that something specific about the ligands causes the difference in TI and FEP results. However, we note that many results for the thrombin target feature similar transformations yet exhibit no significant differences.

**Figure 3:**
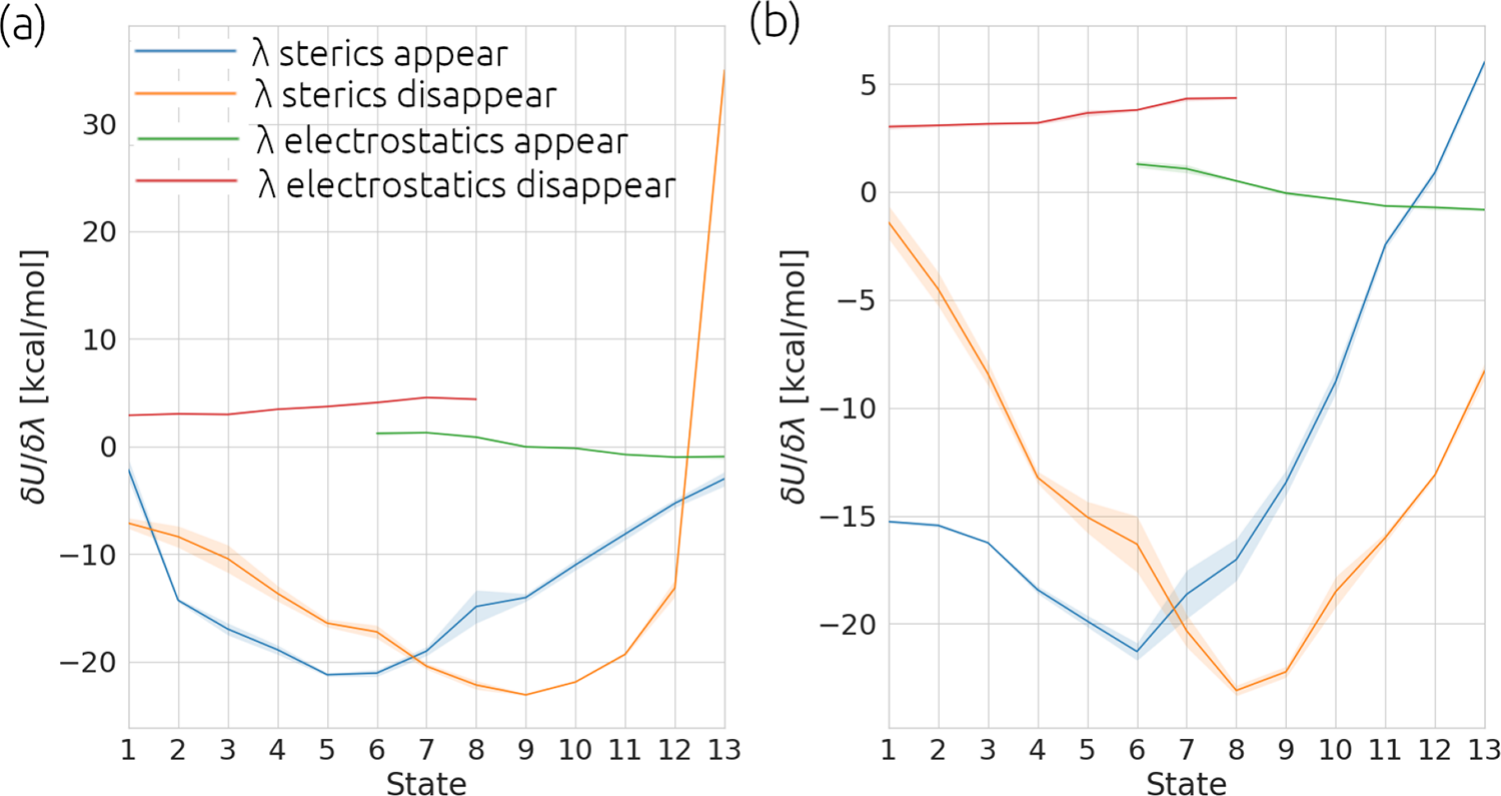
Gradients of potential with respect to λ parameters for transformation l2-l5 simulated with soft-core α = 0.5 (a) and α = 0.7 (b). Shaded regions show the mean ± SEM calculated from five replica calculations in each window.

Without a definitive relation to the specifics of the transformation, the cause of this difference is instead attributed to the behavior of  at the end states for these transformations in the NAMD cases. This can be seen by plotting this gradient for the complex leg of the simulation across all states for the NAMD2 l2-l5 case in Figure 3a. In Figure 3a, we observed a rapid change for the gradient of the potential with respect to the λ parameter, which controls the LJ interactions of the disappearing alchemical region. Rapid changes or excess curvature such as this may result in poor accuracy for numerical integration, and without due care, this is known to be a weakness of the TI method.^49,50^ This rapid change of the gradient is characteristic of all the transformations where we observe differences in the TI and FEP results. Moreover, these rapid changes are lessened or do not exist in the OpenMM case, explaining why no differences are observed.

In this work, the key difference between OpenMM and NAMD methodologies, which pertained to the LJ interactions, lies in the parameters employed in the soft-core potential. The OpenMM method used *c* = 6, while NAMD used *c* = 2, so as such, the OpenMM potential is softer. To test if a softer potential in NAMD can alleviate the difference in the TI and FEP calculations, the selected transformations are repeated using NAMD2 with a soft-core potential with parameters α = 0.7, *a* = 1, *b* = 1, and *c* = 2. Notice that α is modified here because *c* cannot be set by the user in NAMD. Table 4 shows the resulting ΔΔ*G* values for the repeated calculation and the new differences with the equivalent FEP calculation. The results in Table 4 show that there are no remaining significant differences in the TI and FEP results for these transformations. Additionally, it can be seen in Figure 3b that the gradient no longer features a rapid change in the final state. This is consistent with results previously obtained in the literature.^42,49^ It should be noted that the choice of α = 0.7 may not be best in all cases and other choices of soft-core parameters should be considered in general.^42^

**Table 4:** Difference between FEP and TI Results (kcal/mol) for the Two Transformations in Thrombin Target Rerun with NAMD2 with Different Values of the Soft-Core α Parameter^a^

transformation	α = 0.5	α = 0.7
l1-l8	0.40(0.34)	–0.01(0.33)
l2-l5	0.46(0.30)	–0.03(0.30)

a The error provided in parenthesis is calculated by adding the TI and FEP calculation SEM in quadrature.

**Figure 4:**
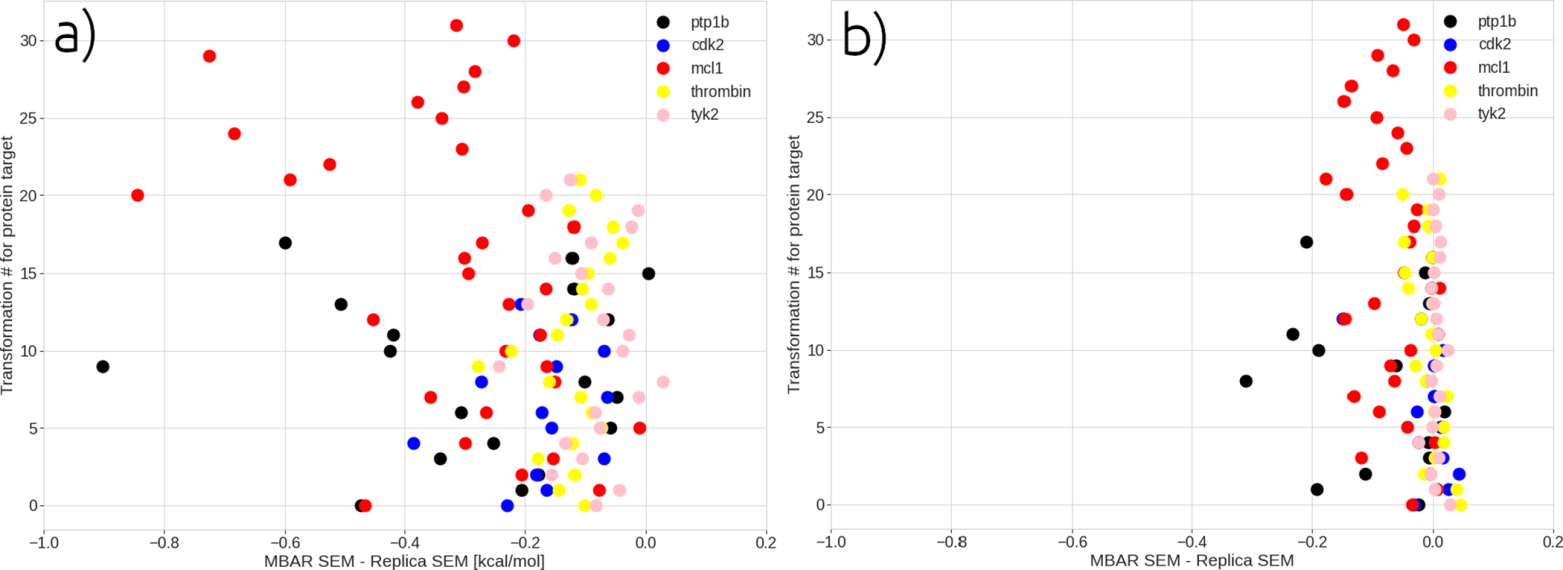
A comparison of the SEM estimated by MBAR from one replica and then averaged over 5 replicas, compared to a “TIES-like” error calculated by computing SEM of the bootstrapping result of 5 replicas. Panels a) and b) show the results for the ligand-protein and ligand only simulations respectively. The y-axis denotes an index assigned to each ligand transformation. This index runs from zero to the total number of transformations minus one, within each protein target across all engines.

In the Methods section, it was noted that there was a caveat in the NAMD2 methodology regarding the lower FEP sampling rate. When TI results from previous work^25^ were reanalyzed with FEP, a sampling rate for the potentials of one-fifth of the rate used to sample the gradient in the TI analysis had to be used. Based on the largely similar absence of differences between FEP and TI in the NAMD2 and NAMD3 cases (where NAMD3 used full sampling) and the ability to treat the small number of differences in NAMD2 case by adjusting the soft-core potential, we conclude that the different sampling rates did not have a significant impact on the difference in TI and FEP results (Figure 4).

#### 4.2.2 Overlap between Alchemical States in FEP Calculations

**Figure 5:**
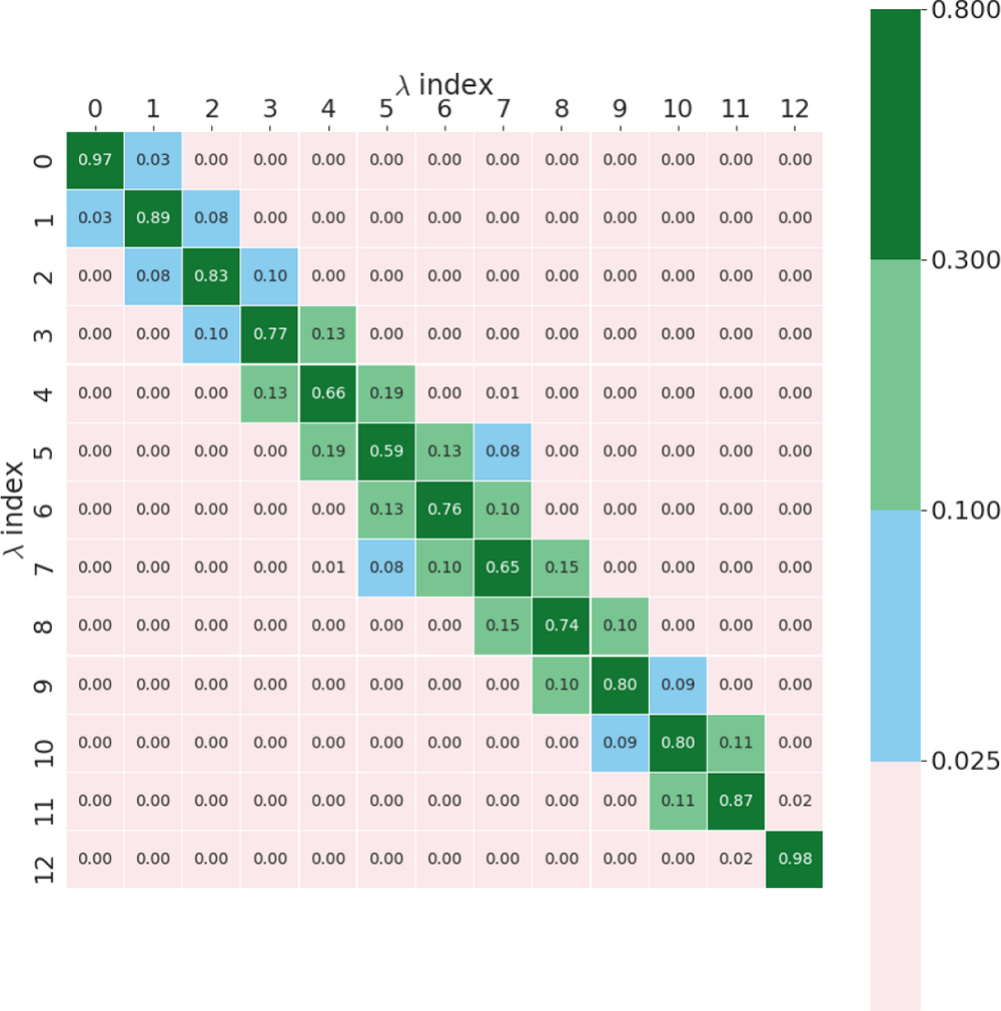
Overlap matrix calculated for the OpenMM MCL1 l12-l35 complex simulation averaged from five replicas.

**Figure 6:**
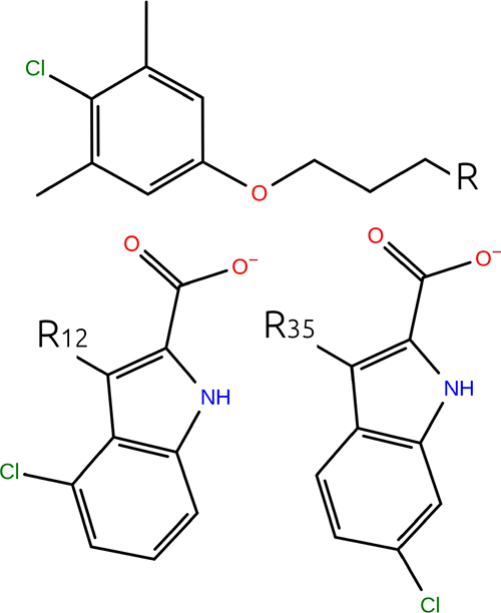
Substituted groups and common substructure for MCL1 transformations l12-l35. Moieties labeled *R_x_* are substituted onto the common substructure at the position denoted by *R*; e.g., swapping *R*_12_ and *R*_35_ is the ligand transformation l12-l35.

**Figure 7:**
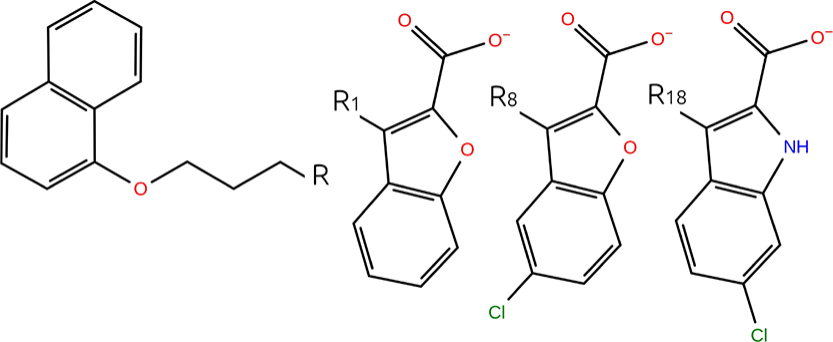
Substituted groups and common substructure for MCL1 transformations l1-l8 and l8-l18. Moieties labeled *R_x_* are substituted onto the common substructure at the position denoted by *R*; e.g., swapping *R*_1_ and *R*_8_ is the ligand transformation l1-l8.

Another difference in the TI and FEP results may stem from the perturbative nature of FEP. If the phase space overlap of alchemical states is small, then the FEP result may be unreliable. A quantitative measure of this overlap of states can be made with an overlap matrix that was computed for all transformations and thermodynamic legs. The overlap matrix is described in detail elsewhere,^45^ but briefly, it is a matrix of rank *K* × *K*, where *K* was previously defined in that work as the number of alchemical states. Each entry in the matrix is the probability that a sample from a given alchemical window λ_*i*_ could have been sampled from some other alchemical window λ_*k*_. For reliable free energy calculations, it has been proposed in previous work that the overlap matrices should be tridiagonal with off-diagonal values greater than 0.03.^43^ When the overlap matrices are averaged across replicas, all but one of the FEP calculations performed in this work satisfied these conditions, and this result is shown in Figure 5. The simulation with the abnormal matrix is the complex leg of an OpenMM simulation for the MCL1 target. The abnormal transformation is named l12-l35; Figure 6 shows this transformation explicitly. If the overlap matrices are not averaged over replicas, there are more instances of results that do not reach the threshold of 0.03, and these all occur for the complex leg of the MCL1 target simulations. Over half of these low overlap cases are for the OpenMM protocol, and six out of eight of the cases are for transformations substantially similar to l12-l35 (see Figure 7 for representative examples of such transformations). Without averaging over replicas, the value of the overlap averaged over all instances failing to reach the 0.03 threshold is 0.02. If the same entries of the overlap matrices are averaged over all replicas, this value increases to 0.05. This further underlines the importance of ensemble simulations in such calculations for ensuring reproducibility of predicted free energies. Despite lower overlap in some cases, this does not manifest itself as a significant difference between the TI and FEP results for these transformations. To show this conclusively, Table 5 exhibits the difference in Δ*G* results in the low-overlap MCL1 case for the complex leg.

**Table 5:** Difference in TI and FEP Complex Δ*G* Result for which Overlap Matrix Exhibits Indication of Low Overlap between Adjacent States^a^

method	transformation	TI-FEP (kcal/mol)
NAMD2	l8-l18	0.09(0.98)
	l1-l8	–0.27(1.15)
	l16-l34	0.47(1.08)
NAMD3	l1-l8	0.61(0.77)
OpenMM	l8-l18	0.19(1.22)
	l1-l8	–0.05(1.06)
	l12-l35	–0.10(1.19)
	l32-l38	–0.50(0.57)

a The error provided in parenthesis is computed by adding error on TI and FEP result in quadrature.

From the overall good agreement we find between the results calculated using the TI and FEP estimators, we remark on the conclusion of previous studies,^51^ which compared Schrödinger’s FEP+ to other TIES-based alchemical methods, revealing significant underestimation of the free energies when using FEP+. In this case, there are several sources of difference in the methodologies, including different force fields and the use of replica exchange with solute tempering 2 (REST2) by FEP+. Since the present study finds good agreement between TI and FEP estimators, it is clear that further work is required to unravel these significant differences. The proprietary nature of FEP+ does not make any such study straightforward, but recent work has shown that REST2 typically degrades results.^51,52^

#### 4.2.3 MBAR Uncertainty Calculated with One-off Simulations

The comparisons between different MD engines and free energy estimators performed in this work could only be made meaningfully when the uncertainty in the binding free energy is accounted for correctly. The results from one-off simulations are not reproducible, and so .only with the proper application of ensemble simulation could such good agreement between the MD engines and free energy estimations compared in this work be found. The application of multiple independent simulations was critical for our error control; similar ideas are found elsewhere in the literature.^33,43,53,54^ If only one-off simulations are performed, errors are consistently underestimated in these calculations. Figure 4 shows this explicitly by comparing the SEM of the analytic MBAR error from five replicas to the “TIES-like” SEM calculated by bootstrapping the results from five replicas. It can be seen that for the ligand simulations in Figure 4b, some systems (TYK2, CDK2, and thrombin) have errors correctly estimated by MBAR, but for PTP1B and MCl1, MBAR consistently underestimates the error. This underestimation is only exacerbated in the complex simulations (Figure 4a), where all systems have their error underestimated by MBAR. This is most likely due to the greater relevance of “rare events” in the complex simulation. Similar findings by Rizzi et al. have concluded “Nevertheless, when sampling is governed by rare events and systematically misses relevant areas of conformational space, data from a single trajectory simply cannot contain sufficient information to estimate the uncertainty accurately.”^41^ It has often been argued that the time series of potentials fed to MBAR should be de-correlated to ensure reliable error estimation.^41^ De-correlation of the time series of potentials, in this case, does not change any of the conclusions. For completeness, we provide an equivalent version of Figure 4 using de-correlated data in the SI (see Figure S1), which demonstrates this conclusively.

### 4.3 Statistical Properties of Relative Free Energy Calculations

#### 4.3.1 Relative Free Energy Distributions

**Figure 8:**
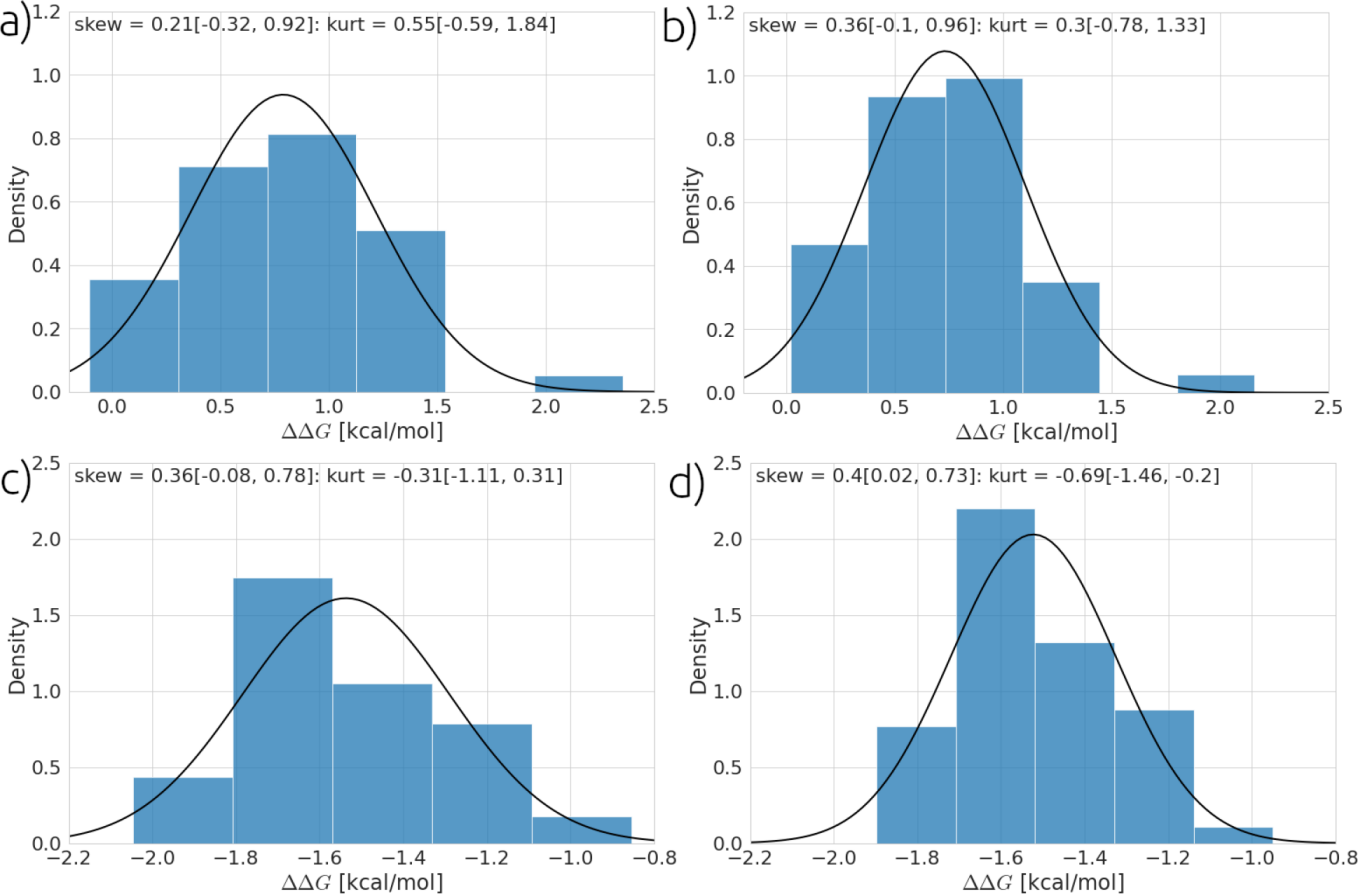
Distribution of the relative binding free energies from for 48 simulations. (a) and (b) show the distribution for thrombin ligand l2-l5 with results estimated by TI and FEP, respectively. (c) and (d) show the distribution for the thrombin ligand 11-l4 with results estimated by TI and FEP, respectively. Parentheses provide 90% bootstrapped confidence intervals on calculation of skewness and excess kurtosis (kurt). The black line shows a Gaussian distribution with the same mean and σ as the plotted data.

**Figure 9:**
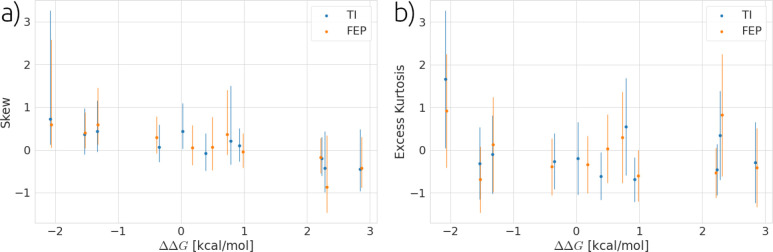
(a) and (b) show the skewness and excess kurtosis for all 11 thrombin ligand transformations examined using both TI and FEP estimators. Error bars are plotted as 90% bootstrapped confidence intervals.

To examine the distribution of calculated binding free energies, we selected the thrombin system and the OpenMM protocol to run larger ensembles of simulations. Forty-eight simulations are run in all 13 λ windows for 4 ns, with all 11 ligands examined for the thrombin target. An analysis for these results is made one replica at a time, and Figure 8 shows examples of the distribution of the relative binding free energies that are found in the results. We plot these results with a calculation of the skewness and excess kurtosis. The skewness characterizes the symmetry of the distribution, and kurtosis is related to the tails of the distribution, where higher values of the kurtosis indicates the presence of a significant number of outliers in the distribution. Here, we report the “excess kurtosis” as kurtosis-3. The excess kurtosis measures the deviation of the kurtosis with respect to the kurtosis one would expect for a Gaussian distribution. Figure 8 shows distributions of the binding free energy for two randomly selected ligand transformations; these distributions are symmetric within error in terms of both skewness and excess kurtosis. If we examine all values of skewness and excess kurtosis as plotted in Figure 9 as a function of the relative binding free energy, it can be seen that although many results look approximately Gaussian, there are distributions at 90% confidence with significant skew and kurtosis. Overall, these results imply the presence of non-normal distributions. This is consistent with previous computational work and recent experimental work, which have reported non-Gaussian distributions for binding free energies.^29,31^

#### 4.3.2 Comparing Long and Large Ensemble Simulations

Previous work using this data set of input transformations and target proteins has demonstrated that an ensemble of five replica simulations using 13 alchemical windows with 4 ns of sampling per window provides a good trade-off of computation cost against accuracy and precision. For completeness, we re-examine this rule of thumb in the context of our work’s larger set of free energy estimators and MD engines. To perform this comparison, 6 ligand transformations are selected from the full set of 54 named in previous work^25^ as l12-l35 and l16-l34 for the MCL1 target, l15-l10 and l15-l16 for the TYK2 target, and l3-l23 and l13-l20 for the PTP1B target. These six transformations are then rerun using all estimators and engines with the same TIES methodology previously discussed but now modified in one of two ways. The first modification is to use 20 sets of 4 ns simulations instead of 5 sets of 4 ns per window, which we call large ensemble runs. The second modification is to use 5 sets of 40 ns runs per window, which we call long runs.

**Figure 10:**
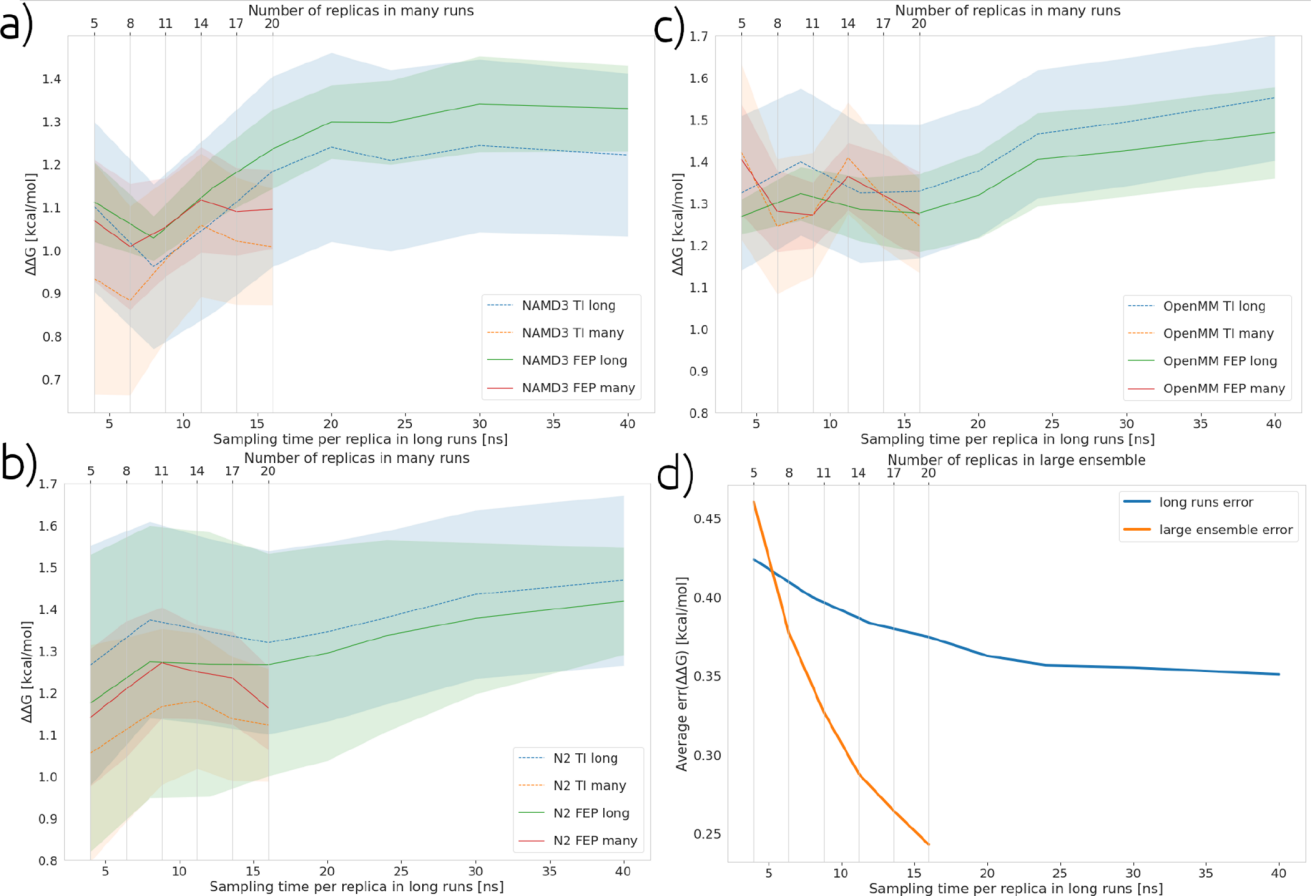
Calculated relative binding free energies for ligand transformation l15-l16 from the target TYK2 (experimental result for this ligand is 0.75 kcal/mol). This figure compares long and large ensemble simulation protocols. (a), (b), and (c) show the results acquired using NAMD3, NAMD2 (N2), and OpenMM, respectively. (d) plots the average statistical uncertainty for all transformations, again comparing long and large ensemble simulation protocols. Shaded regions show the mean ± SEM calculated from five replicas.

In Figure 10, we see a comparison of results collected using the long and large ensemble simulation protocols with one ligand transformation for the TYK2 target. What can be seen from the results in Figure 10 is that even when using less production simulation, the use of many independent and shorter simulations provides similar accuracy and better precision than using fewer and longer simulations. This is a repeated pattern, and Figure 10c shows that the error on the large ensemble runs is much lower than that of the long runs when averaged over all six transformations examined here. Tables 6 and 7 compare the accuracy of large ensemble and long runs and shows that, indeed, averaged over all ligand transformations, the accuracy of these methods is similar despite using less overall simulation time in the large ensemble runs.

**Table 6:** Comparing the TI Accuracy of Large Ensemble Runs Using 20 Replicas of 4 ns and Long Run Using Five Replicas of 40 ns or the Standard TIES Protocols for all Six Ligand Transformations Studied^a^

protocol	property	NAMD3 TI	NAMD2 TI	OpenMM TI
large ensemble runs	MUE	0.58[−0.11, 1.04]	0.63[−0.15, 1.09]	0.60[0.06, 1.05]
	MSE	0.94[−0.75, 1.87]	1.12[−0.97, 2.19]	0.80[−0.38, 1.56]
	RMSD	0.97[0.32, 1.81]	1.06[0.33, 1.89]	0.90[0.38, 1.57]
long runs	MUE	0.70[0.34, 1.03]	0.78[0.19, 1.23]	0.92[0.58, 1.24]
	MSE	0.68[0.08, 1.23]	1.02[−0.25, 1.89]	1.02[0.38, 1.67]
	RMSD	0.83[0.52, 1.28]	1.01[0.51, 1.64]	1.01[0.73, 1.40]
standard TIES	MUE	0.67[0.00, 1.11]	0.56[0.14, 0.90]	0.80[0.21, 1.22]
	MSE	0.99[−0.67, 1.91]	0.55[−0.14, 1.04]	1.07[−0.47, 1.97]
	RMSD	1.00[0.36, 1.71]	0.74[0.37, 1.26]	1.04[0.46, 1.66]

a Properties and 95% confidence intervals, provided in square brackets, are calculated with bootstrapping. Energies are given in kcal/mol.

**Table 7:** Comparing the FEP Accuracy of Large Ensemble Runs Using 20 Replicas of 4 ns and Long Run Using 5 Replicas of 40 ns or the Standard TIES Protocols for all Six Ligand Transformations Studied^a^

protocol	property	NAMD3 FEP	NAMD2 FEP	OpenMM FEP
large ensemble runs	MUE	0.55[−0.09, 0.99]	0.60[−0.02, 1.00]	0.62[0.05, 1.00]
	MSE	0.83[−0.64, 1.64]	0.83[−0.62, 1.59]	0.77[−0.43, 1.47]
	RMSD	0.91[0.31, 1.67]	0.91[0.31, 1.56]	0.88[0.36, 1.49]
long runs	MUE	0.66[0.34, 0.90]	0.64[0.19, 1.02]	0.84[0.55, 1.12]
	MSE	0.55[0.02, 0.93]	0.70[−0.21, 1.30]	0.84[0.33, 1.33]
	RMSD	0.74[0.44, 1.07]	0.84[0.40, 1.35]	0.91[0.67, 1.24]
standard TIES	MUE	0.55[−0.10, 1.00]	0.45[0.02, 0.74]	0.75[0.29, 1.11]
	MSE	0.83[−0.62, 1.64]	0.43[−0.27, 0.83]	0.85[−0.19, 1.51]
	RMSD	0.91[0.31, 1.67]	0.65[0.25, 1.14]	0.92[0.47, 1.42]

a Properties and 95% confidence intervals, provided in square brackets, are calculated with bootstrapping. Energies are given in kcal/mol.

## 5 Conclusions

In this work, 54 ligand transformations for five diverse protein targets: MCL1, PTP1B, TYK2, CDK2, and thrombin have been examined, and relative binding free energy calculations were performed using three MD packages: NAMD2, NAMD3, and OpenMM. The protocols used are built such that the parameters of the protocol that dominates the error in free energy calculations^27^ are matched as closely as possible. Some differences persist between the MD engines, such as the use of diverse soft-core parameters, λ schedules, methods for calculating the TI gradient, and either decoupling or annihilating methods to turn off the alchemical regions. We conclude that while lack of feature parity, even between different revisions of the same MD program, may appear to be a significant obstacle to reproducibility, careful application of RBFE methods can produce results that agree across engines within 0.50 kcal/mol. Differences in individual RBFE calculations can manifest, but our results show no systematic degradation of results for the specific MD engines or free energy estimators that were applied in this work, and all engines were found to be comparably accurate. The correlation between predictions by the different MD engines is very good, the lowest Spearman’s, Pearson’s, and Kendall’s tau correlation coefficients being 0.92, 0.91, and 0.76, respectively.

The agreement that can be achieved between free energy estimators within the same MD engine is even better. It was found that when using proper softcore potential parameters, there was no difference between the TI and FEP results. While such a result has been obtained previously for simple and rigid benchmark molecules in TIP3P water,^42^ we demonstrate it here conclusively using real ligand–protein complexes across multiple MD engines.

Low-phase space overlap between adjacent alchemical states is often quoted as a potential weakness of FEP;^43^ here, we show that for the systems examined and the TIES protocol, this is an insignificant issue. It was possible to heuristically characterize rare instances as exhibiting low overlap, but they had no significant impact on the results. From the 324 relative binding free energy calculations performed in this work, one was identified as having a low overlap: this was l12-l35 in the MCL1 target. However, this low overlap was not found to translate into a difference in the TI and FEP results. Low overlap was found more frequently if analysis was made for “one-off” simulations, but averaging over many replicas eliminated this in all but the one MCL1 case.

While the absence of issues caused by low overlap is a point in favor of FEP and MBAR, we found a negative point in its especially unreliable estimation of error. We compared the SEM of the analytic MBAR error from five replicas to the “TIES-like” SEM calculated by bootstrapping the results from five replicas and demonstrated the MBAR error to be much too small. The MBAR error was in some cases underestimated by up to 0.9 kcal/mol and thus would be inadequate to rank transformations in a drug design campaign. Such underestimations have been observed previously in the literature,^41,51^ and in this work, we demonstrate the underestimation to be consistent and systematic in protein–ligand binding free energy calculations.

With the exception of the TI cases with rapid changes of the gradient in the end states, both TI and FEP methods achieve comparable accuracy and precision for the systems studied in this work, and as such, neither TI nor MBAR is highlighted as preferred. We conclude that there is a clear benefit from using both TI and FEP results in tandem to check the results of the other. While using many MD packages to check the reproducibility of results incurs significantly more cost and may not always be practical, the use of two or more free energy estimators incurs little additional cost and, as shown in this work, can aid in the identification and diagnosis of the alchemical protocol for specific issues causing poor accuracy or precision in the results.^45^

We have also investigated in this work the statistical properties for the distribution of relative binding free energies. Our results for repeating a subset of simulations for 48 replicas allow for the observation of non-Gaussian distributions. This assessment of the distribution was made for all 11 transformations of the thrombin target using the OpenMM protocol developed in this work. The findings of both skewness and kurtosis in the distributions of calculated free energies is consistent with previous computational work and recent experimental work, which have reported non-Gaussian distributions for binding free energies.^29,31^ While this result seems to contradict previous work by Paliwal et al.,^42^ which found free energy distributions to be Gaussian, that work examined much simpler systems of small molecule hydration. In this work, we consider “real-world” molecular systems that are strongly influenced by anharmonic terms (e.g., van der Waals interactions) not performed in a homogeneous environment (e.g., in a protein) and exhibit more than one dominant conformational substrate.^55^ The underlying nonlinearities in the dynamics are what accounts for both the presence of chaos and non-Gaussian statistics. Moreover, we have recently published a paper that shows that experimental binding free energy data indeed display non-normal behavior.^31^ In general, a Gaussian distribution of free energy results can only be assumed for harmonic systems or transformations that can be approximated by linear response theory.^56−58^

The type of distribution relative free energies are sampled from is manifestly important for the accuracy, precision, and uncertainty quantification of RBFE calculations. We have demonstrated this by comparing two extended RBFE protocols, one using a long simulation of 40 ns with five replicas and another with a large ensemble of 20 replicas with 4 ns of sampling. This comparison was made for six transformations examined with all MD engines and estimators considered in this work. The results here show that the use of large ensembles of shorter simulations as compared to smaller ensembles of longer simulations yield comparable accuracy and improved precision, a finding that is true even when using less overall production simulation time in the large ensemble cases.

## Supporting Information Available

All individual results for relative binding free energy calculations performed as well as the input used to generate these results (https://zenodo.org/record/5767275#.YbCZEXX7SV4); (Tables S1–S6) result of the main TIES protocol; (Table S7) result of the long and large ensemble TIES protocols; (Figure S1) equivalent version of Figure 4 plotted using de-correlated data; (Table S8) PDB codes for the proteins used as input to this work; (Table S9) more detailed information for the performance of MD engines at different system sizes (PDF)

## Footnotes

1 https://ucl-ccs.github.io/TIES_MD/

2 https://ccs-ties.org/^29^
