# Supplementary material for: Alchemical Free Energy Estimators and Molecular Dynamics Engines: Accuracy, Precision, and Reproducibility
Source: J Chem Theory Comput. 2022 May 24;18(6):3972–87. doi: 10.1021/acs.jctc.2c00114 (PMC9202356; doi:10.1021/acs.jctc.2c00114)
Supplement: Supplementary file 1 — ct2c00114_si_001.pdf [file ct2c00114_si_001.pdf]

Supplementary Information: Alchemical Free Energy Estimators and Molecular Dynamics Engines: Accuracy, Precision and Reproducibility

Alexander D. Wade,† Agastya P. Bhati,† Shunzhou Wan,† and Peter V. Coveney*,†,‡,¶

†*Centre for Computational Science, Department of Chemistry, University College London, London WC1H 0AJ, UK*

‡*Informatics Institute, University of Amsterdam, Amsterdam 1098XH, The Netherlands*

¶*Centre for Advanced Research Computing, University College London, London UK,*

E-mail: p.v.coveney@ucl.ac.uk

Phone: +44 (0)20 7679 4560

ORCIDs: Alexander D. Wade - 0000-0003-1500-3733 , Agastya P. Bhati - 0000-0003-4539-4819, Peter V. Coveney - 0000-0002-8787-7256

S1 - $\Delta\Delta G$ results: main TIES protocol

In the main body of work we calculate binding $\Delta\Delta G$s using 3 MD and 2 free energy estimators. We present in the main work the statistical properties associated with these calculation i.e. MUE, MSE etc. Here we present the $\Delta\Delta G$ results for all ligand transformation explicitly. Tables S1 - S6 show the results for NAMD2, OpenMM and NAMD3.

Table S1: $\Delta\Delta G$ results calculated using NAMD2 with TI and FEP for targets PTP1B, CDK2 and MCL1. All energies in $kcal/mol$. SEM is standard error of the mean.

**Protein**	**Transformation**	$\Delta\Delta G$ **TI**	$\Delta\Delta G$ **SEM TI**	$\Delta\Delta G$ **FEP**	$\Delta\Delta G$ **SEM FEP**
PTP1B	l10-l12	0.19	0.61	-0.17	0.59
	l13-l20	0.5	0.31	0.45	0.38
	l11-l23	1.75	0.32	1.66	0.32
	l8-l14	1.42	0.46	1.45	0.44
	l1-l2	1.56	0.33	1.52	0.36
	l4-l22	-0.59	0.48	-0.23	0.11
	l19-l3	1.17	0.32	1.29	0.41
	l3-l7	0.15	0.23	-0.03	0.13
	l3-l23	0.77	0.41	0.85	0.43
CDK2	l1q-li9	1.73	0.7	1.63	0.32
	l1q-l17	-0.41	0.29	-0.46	0.23
	l1q-l20	1.72	0.33	1.85	0.26
	l1q-l26	1.34	0.33	1.26	0.15
	l1q-l29	2.11	0.44	1.86	0.49
	l20-l21	-2.26	0.6	-2.14	0.25
	l1q-l21	-1.13	0.25	-1.19	0.26
MCL1	l2-l4	3.03	0.4	3.08	0.56
	l6-l41	0.16	0.34	0.2	0.17
	l3-l5	1.05	0.27	1.18	0.28
	l3-l16	2.98	0.3	3.32	0.29
	l16-l34	-0.65	1	-0.23	0.46
	l12-l35	2.09	1.23	2.09	0.25
	l2-l32	1.5	0.36	1.88	0.38
	l32-l42	1.28	0.47	1.28	0.48
	l38-l42	-3.28	0.35	-3.55	0.27
	l32-l38	3.79	0.4	3.95	0.31
	l39-l42	-2.45	0.28	-2.25	0.34
	l18-l39	-3.3	0.89	-2.81	0.33
	l1-l8	-0.08	1.02	-0.22	0.65
	l8-l18	3.76	0.94	3.8	0.44
	l17-l9	-0.05	0.28	-0.2	0.24
	l13-l17	0.77	0.38	0.79	0.38

Table S2: $\Delta\Delta G$ results calculated using NAMD2 with TI and FEP for targets TYK2 and thrombin. All energies in $kcal/mol$. SEM is standard error of the mean.

**Protein**	**Transformation**	$\Delta\Delta G$ **TI**	$\Delta\Delta G$ **SEM TI**	$\Delta\Delta G$ **FEP**	$\Delta\Delta G$ **SEM FEP**
TYK2	l1-l3	0.12	0.14	0.27	0.13
	l1-l6	-1.33	0.11	-1.37	0.09
	l6-l11	2.06	0.45	2.27	0.25
	l6-l10	-1.11	0.14	-1.1	0.17
	l15-l16	1.07	0.23	0.94	0.22
	l1-l8	-1.03	0.11	-1.08	0.14
	l5-l16	0.2	0.18	0.29	0.13
	l1-l15	0.08	0.08	0.09	0.04
	l15-l6	-1.47	0.11	-1.44	0.04
	l1-l10	-2.18	0.22	-2.09	0.33
	l15-l10	-2.51	0.12	-2.47	0.09
thrombin	l8-l1	0.83	0.29	0.87	0.17
	l1-l8	-0.37	0.27	-0.77	0.21
	l6-l7	-0.52	0.24	-0.61	0.19
	l3-l5	2.4	0.28	2.36	0.25
	l5-l6	-1.99	0.2	-2.18	0.19
	l1-l4	-1.58	0.38	-1.62	0.12
	l2-l5	1.05	0.26	0.59	0.15
	l4-l11	1.85	0.31	2.17	0.15
	l7-l3	0.2	0.31	0.33	0.23
	l1-l9	1.15	0.27	1.04	0.35
	l4-l10	2.18	0.3	2.34	0.3

Table S3: $\Delta\Delta G$ results calculated using OpenMM with TI and FEP for targets PTP1B, CDK2 and MCL1. All energies in $kcal/mol$. SEM is standard error of the mean.

**Protein**	**Transformation**	$\Delta\Delta G$ **TI**	$\Delta\Delta G$ **SEM TI**	$\Delta\Delta G$ **FEP**	$\Delta\Delta G$ **SEM FEP**
PTP1B	l10-l12	0.45	0.62	0.3	0.95
	l13-l20	-0.19	0.55	-0.19	0.51
	l11-l23	0.26	0.46	0.38	0.54
	l8-l14	0.83	0.28	0.83	0.11
	l1-l2	0.99	0.44	0.78	0.54
	l4-l22	0.09	0.3	0.03	0.14
	l19-l3	0.84	0.31	0.89	0.07
	l3-l7	0.33	0.28	0.29	0.15
	l3-l23	0.49	0.55	0.39	0.69
CDK2	l1q-li9	1.78	0.2	1.77	0.09
	l1q-l17	-1.56	0.22	-1.39	0.3
	l1q-l20	1.72	0.17	1.69	0.18
	l1q-l26	1.28	0.17	1.36	0.1
	l1q-l29	2.34	0.23	2.35	0.22
	l20-l21	-3.29	0.31	-3.4	0.24
	l1q-l21	-1.34	0.26	-1.33	0.24
MCL1	l2-l4	3	0.43	3.24	0.34
	l6-l41	-0.13	0.31	-0.19	0.31
	l3-l5	1.03	0.26	0.96	0.16
	l3-l16	2.58	0.32	2.59	0.23
	l16-l34	0.61	0.78	0.53	0.93
	l12-l35	2.82	1.02	2.46	0.71
	l2-l32	2.89	0.56	2.94	0.57
	l32-l42	1.2	0.39	1.14	0.34
	l38-l42	-1.64	0.51	-1.66	0.73
	l32-l38	3.27	0.46	2.88	0.41
	l39-l42	-0.79	0.37	-0.73	0.45
	l18-l39	-1.33	0.81	-1.52	0.41
	l1-l8	0.45	1.02	0.29	0.37
	l8-l18	2.73	0.95	2.63	0.8
	l17-l9	-0.39	0.24	-0.59	0.25
	l13-l17	0.75	0.57	0.71	0.35

Table S4: $\Delta\Delta G$ results calculated using OpenMM with TI and FEP for targets TYK2 and thrombin. All energies in $kcal/mol$. SEM is standard error of the mean.

**Protein**	**Transformation**	**$\Delta\Delta G$ TI**	**$\Delta\Delta G$ SEM TI**	**$\Delta\Delta G$ FEP**	**$\Delta\Delta G$ SEM FEP**
TYK2	l1-l3	0.33	0.08	0.36	0.04
	l1-l6	-1.31	0.1	-1.34	0.09
	l6-l11	1.41	0.29	1.49	0.23
	l6-l10	-1.38	0.09	-1.39	0.08
	l15-l16	1.42	0.21	1.4	0.13
	l1-l8	-0.86	0.19	-0.98	0.18
	l5-l16	0.6	0.26	0.57	0.11
	l1-l15	-0.16	0.06	-0.15	0.04
	l15-l6	-1.05	0.08	-1.01	0.03
	l1-l10	-2.45	0.23	-2.56	0.2
	l15-l10	-2.59	0.13	-2.63	0.15
thrombin	l8-l1	-0.03	0.18	0.13	0.18
	l1-l8	-1.47	0.13	-1.4	0.16
	l6-l7	-0.53	0.15	-0.5	0.12
	l3-l5	2.16	0.23	2.14	0.15
	l5-l6	-2.61	0.24	-2.36	0.14
	l1-l4	-1.48	0.1	-1.49	0.08
	l2-l5	0.5	0.16	0.56	0.09
	l4-l11	2.26	0.11	2.33	0.08
	l7-l3	0.41	0.18	0.52	0.15
	l1-l9	0.8	0.15	0.89	0.13
	l4-l10	2.79	0.17	2.82	0.14

Table S5: $\Delta\Delta G$ results calculated using NAMD3 with TI and FEP for targets PTP1B, CDK2 and MCL1. All energies in $kcal/mol$. SEM is standard error of the mean.

**Protein**	**Transformation**	$\Delta\Delta G$ **TI**	$\Delta\Delta G$ **SEM TI**	$\Delta\Delta G$ **FEP**	$\Delta\Delta G$ **SEM FEP**
PTP1B	l10-l12	1.12	0.46	1.16	0.38
	l13-l20	0.24	0.37	0.39	0.24
	l11-l23	1.16	0.41	0.97	0.38
	l8-l14	1.11	0.35	1.14	0.27
	l1-l2	2.42	0.35	2.57	0.42
	l4-l22	-1.18	0.39	-0.57	0.13
	l19-l3	0.91	0.29	0.96	0.23
	l3-l7	-0.35	0.23	-0.35	0.16
	l3-l23	1.37	0.36	1.44	0.33
CDK2	l1q-li9	1.43	0.47	1.54	0.15
	l1q-l17	-0.85	0.25	-0.80	0.22
	l1q-l20	1.78	0.41	1.75	0.11
	l1q-l26	1.06	0.24	1.15	0.09
	l1q-l29	2.85	0.36	2.83	0.23
	l20-l21	-2.94	0.34	-2.92	0.36
	l1q-l21	-1.23	0.26	-1.27	0.10
MCL1	l2-l4	3.61	0.33	3.63	0.41
	l6-l41	0.43	0.30	0.43	0.18
	l3-l5	0.79	0.26	0.94	0.20
	l3-l16	2.99	0.26	3.36	0.08
	l16-l34	0.76	0.78	0.87	0.26
	l12-l35	2.75	0.89	2.40	0.37
	l2-l32	1.73	0.34	1.76	0.26
	l32-l42	1.75	0.55	1.70	0.24
	l38-l42	-3.70	0.34	-3.84	0.14
	l32-l38	5.30	0.41	4.95	0.36
	l39-l42	-2.05	0.33	-2.23	0.34
	l18-l39	-2.12	0.66	-2.02	0.38
	l1-l8	-1.82	0.71	-1.21	0.29
	l8-l18	3.03	0.86	2.92	0.38
	l17-l9	-0.37	0.22	-0.55	0.09
	l13-l17	-0.18	0.29	-0.12	0.14

Table S6: $\Delta\Delta G$ results calculated using NAMD3 with TI and FEP for targets TYK2 and thrombin. All energies in $kcal/mol$. SEM is standard error of the mean.

**Protein**	**Transformation**	**$\Delta\Delta G$ TI**	**$\Delta\Delta G$ SEM TI**	**$\Delta\Delta G$ FEP**	**$\Delta\Delta G$ SEM FEP**
TYK2	l1-l3	0.34	0.17	0.22	0.05
	l1-l6	-1.58	0.11	-1.65	0.05
	l6-l11	1.85	0.26	1.90	0.13
	l6-l10	-1.41	0.13	-1.35	0.08
	l15-l16	1.38	0.24	1.36	0.18
	l1-l8	-1.03	0.12	-1.02	0.04
	l5-l16	0.07	0.17	0.06	0.07
	l1-l15	-0.20	0.09	-0.22	0.03
	l15-l6	-1.59	0.10	-1.49	0.03
	l1-l10	-2.71	0.23	-2.67	0.11
	l15-l10	-3.20	0.14	-3.14	0.14
thrombin	l8-l1	0.61	0.29	0.84	0.23
	l1-l8	-0.34	0.30	-0.70	0.25
	l6-l7	-0.82	0.21	-0.58	0.29
	l3-l5	3.40	0.25	3.25	0.05
	l5-l6	-2.34	0.22	-2.36	0.05
	l1-l4	-0.87	0.27	-1.26	0.10
	l2-l5	1.98	0.22	1.24	0.18
	l4-l11	1.99	0.24	2.30	0.19
	l7-l3	0.71	0.24	0.62	0.12
	l1-l9	1.32	0.22	1.34	0.09
	l4-l10	2.89	0.27	3.01	0.25

S2 - $\Delta\Delta G$ results: extended TIES protocol

Calculations are performed in this work using modified TIES protocols of 20 replicas of 4 ns and 5 replicas of 40 ns. Here we present the results of these calculations explicitly for each ligand transformation in table S7.

S3 - Single Replica Errors vs Many Replica Errors

In the main body of this work a comparison is drawn between the error calculated by MBAR from one-off simulations compared to errors calculated by bootstrapping the results from many replicas. We note that it is often claimed that the error estimation in MBAR can be improved by feeding it a decorrelated time series of potentials. Therefore we repeat the analysis of the main paper, comparing MBAR and many replica errors but now decorrelating the data before making the MBAR estimate. Figure S1 shows this result, and clearly demonstrates that MBAR still underestimates the errors compared to using many replicas.

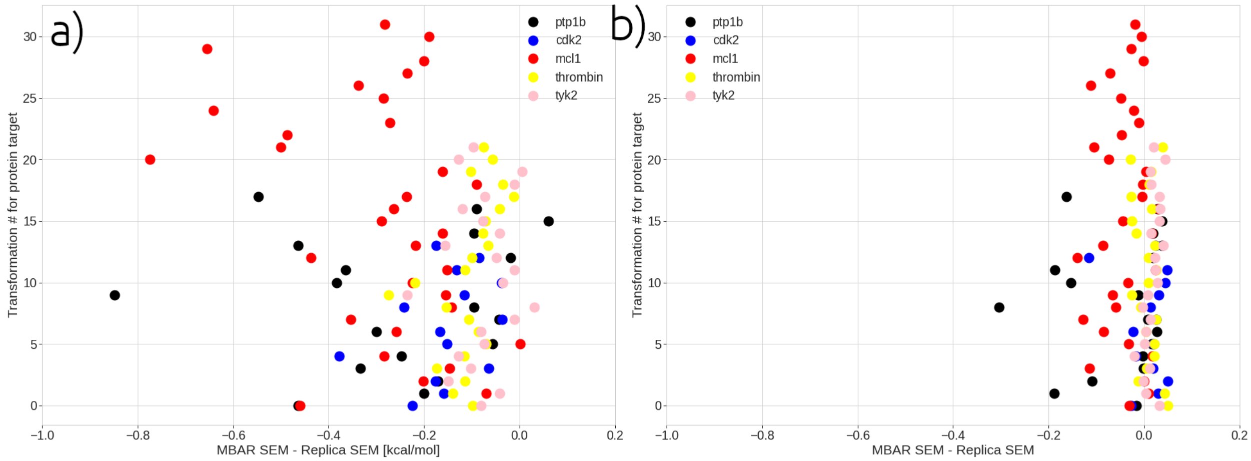

Figure S1: Comparison of MBAR SEM estimated from one replica (calculated with decorrelated potentials), then averaged over 5 replicas, compared to "TIES like" error calculate by computing SEM of bootstrapping result of 5 replicas. Panels a) and b) show the results for the protein-ligand and ligand only simulations respectively. The x-axis denotes an index assigned to each ligand transformation. This index runs from zero to the total number of transformations minus one, within each protein target across all engines.

Table S7: $\Delta\Delta G$ results when using modified TIES method with large ensembles (LE) and long runs (LR). Errors are calculated using standard TIES protocol described in main body of work. All energies in $kcal/mol$. Error calculated is SEM (standard error of the mean.)

**engine**	**method**	**transformation**	**LE $\Delta\Delta G$**	**error LE $\Delta\Delta G$**	**LR $\Delta\Delta G$**	**error LR $\Delta\Delta G$**
NAMD3	TI	l13-l20	0.82	0.18	0.38	0.26
		l3-l23	0.90	0.19	0.61	0.31
		l16-l34	0.27	0.45	1.57	1.11
		l12-l35	2.90	0.43	2.03	0.54
		l15-l16	1.00	0.14	1.22	0.19
		l15-l10	-3.02	0.07	-2.71	0.12
	FEP	l13-l20	0.78	0.13	0.34	0.20
		l3-l23	0.95	0.17	0.56	0.26
		l16-l34	0.27	0.19	1.21	0.25
		l12-l35	2.73	0.28	1.94	0.34
		l15-l16	1.06	0.09	1.34	0.09
		l15-l10	-3.01	0.06	-2.72	0.17
NAMD2	TI	l13-l20	0.43	0.17	0.5	0.32
		l3-l23	1.01	0.2	0.71	0.32
		l16-l34	0.65	0.59	1.46	1.08
		l12-l35	3.15	0.55	2.65	0.81
		l15-l16	1.12	0.13	1.47	0.2
		l15-l10	-2.65	0.07	-2.66	0.11
	FEP	l13-l20	0.42	0.15	0.6	0.32
		l3-l23	1.05	0.22	0.82	0.14
		l16-l34	0.8	0.31	1.22	0.5
		l12-l35	2.73	0.33	2.3	0.38
		l15-l16	1.16	0.1	1.42	0.13
		l15-l10	-2.65	0.06	-2.66	0.12
OpenMM	TI	l13-l20	-0.24	0.26	-0.78	0.38
		l3-l23	0.91	0.3	0.36	0.41
		l16-l34	0.41	0.49	1.06	0.42
		l12-l35	2.55	0.52	2.12	0.8
		l15-l16	1.24	0.11	1.55	0.15
		l15-l10	-2.43	0.06	-2.69	0.07
	FEP	l13-l20	-0.09	0.21	-0.62	0.42
		l3-l23	0.77	0.36	0.46	0.66
		l16-l34	0.44	0.51	1.13	0.24
		l12-l35	2.53	0.36	1.94	0.51
		l15-l16	1.27	0.1	1.47	0.11
		l15-l10	-2.49	0.06	-2.68	0.08

S4 - Input system

Accompanied with this written SI is a zip file of all the parameterized systems used as input to this study https://zenodo.org/record/5767275#.YbCZEXX7SV4. The folders in this zip are structured in the hierarchy protein, transformation, alchemcial leg, input. In the input folder there is the pdb file containing structural information, a AMBER prmtop containing parameter information and for the ligand-protien leg there are a constraints.pdb file which list the constraints used during minimization. The codes for these pdbs as listed on the protein data bank are provided in table S8

Table S8: PDB codes for systems used as input to this study

**Protein**	**CDK2**	**MCL1**	**PTP1B**	**Thrombin**	**TYK2**
PDBID	1H1Q	4HW3	2QBS	2ZFF	4GIH

S5 - Performance

Here we examine more data for the performance NAMD3, NAMD2 and OpenMM. All NAMD3 and OpenMM performance data referenced here were collected from Summit ORNL using Nvidia V100s and the MD protocols referenced in sections 7 and 6 above. NAMD2 performance information was collected from ARCHER in 2017 and thus can be considered out of date but is included here from completeness.

Table S9: ns/day performance for protein systems. Data is provided for the larger ligand-protein (Complex) system as well as the smaller solvated ligand (Solvent) systems.

**Complex**	**Protein**	**CDK2**	**MCL1**	**PTP1B**	**Thrombin**	**TYK2**
	Number atoms	74449	33837	56033	59173	58245
	NAMD3[ns/day]	97	131	99	97	97
	OpenMM[ns/day]	69	116	70	66	69
	NAMD2[ns/day]	13	18	12	12	17
**Solvent**	**Protein**	**CDK2**	**MCL1**	**PTP1B**	**Thrombin**	**TYK2**
	Number atoms	7803	8397	8507	8859	7869
	NAMD3[ns/day]	152	188	139	178	150
	OpenMM[ns/day]	244	220	287	273	263
	NAMD2[ns/day]	42	28	22	30	26
